# The Bilaterian Head Patterning Gene *six3/6* Controls Aboral Domain Development in a Cnidarian

**DOI:** 10.1371/journal.pbio.1001488

**Published:** 2013-02-19

**Authors:** Chiara Sinigaglia, Henriette Busengdal, Lucas Leclère, Ulrich Technau, Fabian Rentzsch

**Affiliations:** 1 Sars Centre for Marine Molecular Biology, University of Bergen, Bergen, Norway; 2 Department of Molecular Evolution and Development, Centre of Organismal Systems Biology, Faculty of Life Sciences, University of Vienna, Vienna, Austria; University of California Berkeley, United States of America

## Abstract

The origin of the bilaterian head is a fundamental question for the evolution of animal body plans. The head of bilaterians develops at the anterior end of their primary body axis and is the site where the brain is located. Cnidarians, the sister group to bilaterians, lack brain-like structures and it is not clear whether the oral, the aboral, or none of the ends of the cnidarian primary body axis corresponds to the anterior domain of bilaterians. In order to understand the evolutionary origin of head development, we analysed the function of conserved genetic regulators of bilaterian anterior development in the sea anemone *Nematostella vectensis*. We show that orthologs of the bilaterian anterior developmental genes *six3/6*, *foxQ2*, and *irx* have dynamic expression patterns in the aboral region of *Nematostella*. Functional analyses reveal that *NvSix3/6* acts upstream of *NvFoxQ2a* as a key regulator of the development of a broad aboral territory in *Nematostella*. *NvSix3/6* initiates an autoregulatory feedback loop involving positive and negative regulators of FGF signalling, which subsequently results in the downregulation of *NvSix3/6* and *NvFoxQ2a* in a small domain at the aboral pole, from which the apical organ develops. We show that signalling by *NvFGFa1* is specifically required for the development of the apical organ, whereas *NvSix3/6* has an earlier and broader function in the specification of the aboral territory. Our functional and gene expression data suggest that the head-forming region of bilaterians is derived from the aboral domain of the cnidarian-bilaterian ancestor.

## Abstract 2

Characterization of bilaterian head patterning genes in a cnidarian reveals a key role for *six3/6* in aboral domain development and provides new insight into the evolutionary origin of head development.

## Author Summary

The evolutionary origin of head development is a fundamental question for understanding the evolution of animal body plans. Bilaterally symmetrical animals (Bilaterians) have an anterior-posterior (head-to-tail) axis, whose anterior end is usually characterized by a nervous system centralization, the brain. This region is often associated with a distinct structure, the head, and its development is regulated by a set of conserved transcription factors and signalling molecules. Bilaterians evolved from an ancestor shared with cnidarians (corals, sea anemones, jellyfish), but brain-like structures are absent in cnidarians, although they have an obvious oral-aboral axis. Cnidarian larvae move with the aboral pole forward, but as adult polyps this pole is anchored to the ground, while the oral end is used for feeding. It is unclear whether one of the termini of cnidarians corresponds to the bilaterian head-forming region. We show here that in the sea anemone *Nematostella vectensis* genes regulating bilaterian head development are expressed at the larval aboral pole and that a key anterior developmental gene, *six3/6*, controls the development of the aboral pole. These findings support the hypothesis that the anterior, head-forming, region of bilaterians and the aboral region of cnidarians derived from the same domain of their last common ancestor and are therefore homologues.

## Introduction

The head of bilaterians develops at the anterior end of the anterior-posterior body axis and is characterized by an accumulation of interconnected nerve cells, the brain. The polarity of the anterior-posterior (A-P) axis is in most bilaterians prefigured by the animal–vegetal axis of the oocyte, with the head forming anterior end developing from the animal hemisphere and the site of gastrulation from the vegetal hemisphere [1]–[3].

Comparative work in bilaterians has revealed highly conserved molecular mechanisms involved in the patterning of the anterior-posterior axis. Orthologous *hox* genes have been shown to have staggered anterior expression borders, providing a unique signature of *hox* gene expression for consecutive domains along the A-P axis. However, while *hox* genes are essential regulators of axial patterning, their expression in most bilaterians does not extend to the anterior-most part of the embryo, where the head will form (e.g., [4]–[8]).

The anterior, *hox*-free region of bilaterian embryos gives rise to head and brain structures and an important regulator of its development is the conserved homeodomain transcription factor *six3*. The anterior expression of *six3* is conserved in the three clades of bilaterians: deuterostomes, ecdysozoans, and lophotrochozoans [9]–[18]. Knockdown of *six3* function in sea urchin results in loss of the anterior/apical plate, *six3* RNAi in the beetle *Tribolium castaneum* leads to failure of anterior head development [11],[19], and *six3* knockout mice lack almost the entire forebrain [20], suggesting that *six3* regulated anterior development already in the last common ancestor of all bilaterians.

A second well-conserved marker for anterior-most territories is the forkhead domain transcription factor *foxQ2*, which functions downstream of *six3* in the development of the anterior/apical domain of sea urchins [11],[21] and is expressed at the anterior tip of the cephalochordate *Branchiostoma floridae* [22], the brachiopod *Terebratalia transversa* [18], the arthropod *Drosophila melanogaster* [23], and at the aboral pole of the cnidarian *Clytia hemispherica* [24].

The posterior pole of a wide range of bilaterians is characterised by the expression of secreted signalling molecules of the *wnt* family. The posterior expression of canonical *wnt* genes results in the nuclear accumulation of β-catenin and determines the site of gastrulation [25]–[32].

Despite the advanced understanding of the mechanisms that pattern the anterior-posterior axis and position the head of bilaterians, the evolutionary origins of axial patterning and head formation are not clear. As the sister group to Bilateria, cnidarians are an essential outgroup to understand the origin of the bilaterian anterior-posterior axis. Cnidarians (sea anemones, corals, jellyfish) do not have a brain-like centralization of the nervous system, but they possess a clear oral-aboral axis [33]. This axis is commonly assumed to be related to the anterior-posterior axis of bilaterians, but there is no consensus as to whether one of the poles of the cnidarian axis corresponds to the anterior, head-forming pole of bilaterians. Competing hypotheses about the relation of the cnidarian primary body axis to the anterior pole of bilaterians suggest that either the oral or the aboral pole of cnidarians is homologous to the bilaterian anterior pole or that there is no homologous region in cnidarians. Similar to many ciliated bilaterian larvae, some anthozoan cnidarian planulae swim with an apical tuft, located opposite to the gastrulation site, pointing forward. These observations have led to the interpretation that the aboral pole of cnidarians corresponds to the anterior pole of bilaterians (e.g., [34]–[36]). In direct contrast, observations from comparative embryology motivated the hypothesis that the oral pole of cnidarians corresponds to the anterior pole of bilaterians [3]. In cnidarians, gastrulation occurs in the domain that is derived from the animal pole of the fertilized egg and the blastopore becomes the only opening of the organism, whereas bilaterians gastrulate from the vegetal pole, but their mouth and anterior nervous system form from the animal domain [3],[37]–[42]. This fundamental embryological difference lead to the hypothesis that in stem bilaterians, determinants of the gastrulation site changed from the animal to the vegetal pole, while determinants of mouth and brain development remained at the animal pole, meaning that the oral pole of cnidarians corresponds to the anterior pole of bilaterians [3]. In a third hypothesis, the absence of nervous system centralization and the incongruent expression of orthologs of some bilaterian anterior patterning genes have been interpreted to suggest that the anterior region of bilaterians has no equivalent in cnidarians [43] (see also [34],[44] for reviews).

The expression patterns of homologs of bilaterian axial patterning genes in cnidarians have not provided support for any of the above-mentioned scenarios. *Hox* genes have frequently been used to understand axial patterning in cnidarians; however, since these genes are not expressed at the anterior-most end of bilaterians, they do not allow the identification of a putative equivalent of the head-forming region in cnidarians. Moreover, anterior group *hox* genes have been found to be expressed either at the aboral pole (*Clytia*, *Eleutheria*, *Podocoryne*; [45]–[47]) or at the oral pole (*Nematostella*; [48]) of cnidarian planulae.

Expression of cnidarian *Wnt* ligands is strongly correlated with the gastrulation site, both in species that gastrulate by invagination and by unipolar ingression [49]–[51]. Functional studies have shown that canonical Wnt/β-catenin signalling is required for proper axial patterning by promoting oral fates in *Hydractinia*, *Clytia*, and *Hydra* [50]–[53], and it has been shown to promote endoderm formation in *Nematostella* [54],[55]. These data suggest that a *Wnt*-expressing signalling centre was already present at the gastrulation site of the last common ancestor of cnidarians and bilaterians. This observation is often taken as evidence that the site of gastrulation in cnidarians identifies their posterior pole, as in bilaterians. However, even if this holds true, it remains unclear whether the opposite, aboral pole is developmentally equivalent to the bilaterian anterior pole (see above).

Attempts to identify a conserved role of bilaterian head development genes in cnidarian development have so far been unsuccessful. These attempts were based on a limited number of genes, and their inconsistent expression patterns [43],[56]–[58] do not rule out that a core set of bilaterian anterior patterning genes with a conserved function in axial patterning exists in cnidarians.

In the present study we show that such a conserved set of bilaterian anterior development genes regulates aboral pole development in the sea anemone *Nematostella vectensis*. *Nematostella* gastrulates by invagination, forming a free swimming planula that carries a ciliated apical organ at the aboral pole. The planula settles on the aboral pole and develops into a sessile polyp [40],[59]–[61]. We analysed the role of orthologs of two conserved bilaterian anterior development genes, *NvSix3/6* and *NvFoxQ2a*, and show that they are involved in the development of the aboral territory in *Nematostella* by establishing an autoregulatory FGF feedback loop that leads to the patterning of the aboral territory into distinct domains. Our functional and expression data suggest that the bilaterian head is derived from the aboral region of the last common ancestor of cnidarians and bilaterians.

## Results

### Bilaterian Head Genes Are Expressed in the Aboral Domain of Developing *Nematostella*

Recent gene expression analyses in bilaterian embryos have identified *six3* and *foxQ2* as highly conserved developmental markers of anterior/apical territories (Figure S1 and Introduction). Moreover, functional studies revealed an essential role for *six3* in anterior development in sea urchins, vertebrates, and the arthropod *Tribolium* [11],[19],[20], indicating that *six3* likely played this role in the common ancestor to all Bilateria.

**Figure 1:**
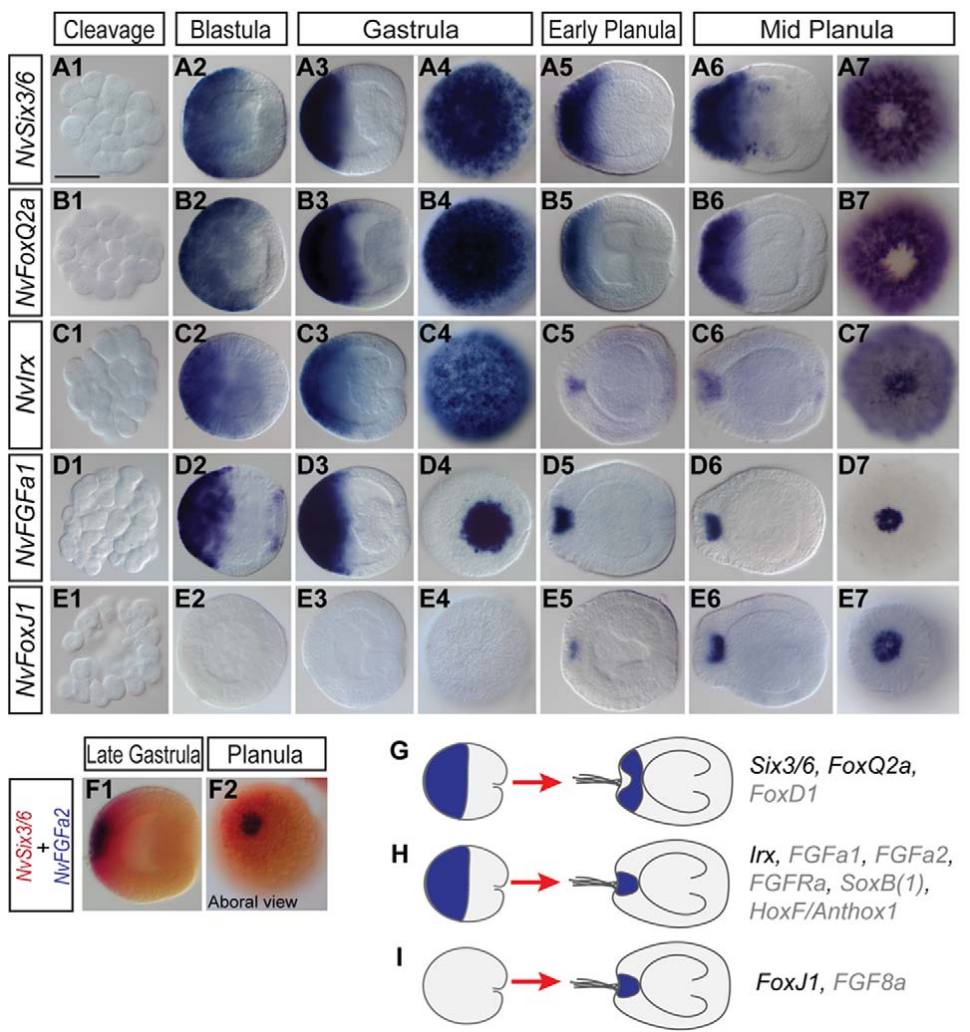
Bilaterian anterior marker genes are expressed at the aboral pole of *Nematostella.* (A1–E7) In situ hybridizations, probes are indicated on the left; developmental stages above the images. (A–E2, A–E3, A–E5, and A–E6) are lateral views, from planula stage on with the aboral pole to the left. At the blastula stage, embryos are oriented with the assumption that the staining is continuous between the blastula and gastrula stage. Note that the future oral and aboral sides cannot be distinguished morphologically prior to gastrulation. (A–E4 and A–E7) are aboral views. Expression domains of *NvSix3/6*, *NvFoxQ2a*, *NvIrx*, and *NvFGFa1* are highly similar until the gastrula stage and then segregate into mutually exclusive domains. *NvFoxJ1* expression becomes detectable only after gastrulation. (F1 and F2) Double in situ hybridization with *NvSix3/6* (red) and *NvFGFa2* (blue) probes, showing the early restriction of the *NvFGFa2* expression from the gastrula stage on. (G–I) Schematic representation of the three types of aboral expression patterns: (G) “ring genes,” (H) “spot genes,” and (I) “late genes.” Synexpression groups are indicated on the right side; genes that have been described previously are shown in gray. Note that *NvSoxB(1)*, *NvHoxF/Anthox1*, and *NvFGF8a* have additional expression domains not indicated in the cartoon.

The *Nematostella* genome contains a single gene of the *six3/6* group and four *foxQ2* genes [62]. In situ hybridization revealed that *NvSix3/6* and one of the *Nematostella foxQ2* genes, *NvFoxQ2a*, display regionally restricted expression patterns: from the blastula stage on, they are expressed in a broad domain on one side of the embryo (Figure 1A1 and A2, 1B1 and B2), which can be identified as the aboral side following the onset of gastrulation (Figure 1A3 and A4, 1B3 and 4). From the mid-planula stage, the expression is excluded from a small spot-like domain at the aboral pole, preceding the appearance of the long cilia of the apical tuft (Figure 1A5–7, 1B5–7). Similar expression dynamics have been described for *NvFoxD1* [63], a gene that is also expressed in the anterior/apical domain of the sea urchin larva and in the mouse forebrain [21],[64]. In addition, *NvSix3/6* is expressed in a small number of scattered cells in both endo- and ectoderm of the planula, which by morphology resemble sensory cells (Figure S2).

Like *NvSix3/6* and *NvFoxQ2a*, the single *Nematostella* ortholog of the bilaterian anterior marker gene *iroquois/irx* [9],[16],[62],[65]–[70] is expressed in a broad aboral domain at the blastula and gastrula stages. However, after gastrulation, the expression of *NvIrx* becomes restricted to a small spot at the aboral pole, matching the site from which *NvSix3/6* and *NvFoxQ2a* are excluded (Figure 1C1–C7). The expression dynamics of *NvIrx* are very similar to that of several previously described genes that are expressed at the aboral pole. These include the FGF signalling components *NvFGFa1*(Figure 1D1–D7), *NvFGFa2*, *FGFRa*, and the transcription factors *NvHoxF/Anthox1* and *NvSoxB(1)*, a *soxB* gene of unresolved orthology to *soxB1* or *B2* subgroups (*NvSoxB1* in [63], [48],[63],[71]–[73], and Figure S3).

Single and double in situ hybridizations show that the restriction of *NvFGFa1* and *NvFGFa2* expression precedes the occurrence of the gap in the expression of *NvSix3/6* and *NvFoxQ2a* (Figure 1F1–F2 and unpublished data).

The apical sense organ, which develops at the aboral pole, consists of a heterogenous group of cells, most of which carry the long cilia that constitute the apical tuft. *FoxJ1* is an important regulator of cilia formation in vertebrates [74], and it has been shown to be expressed in the apical tuft of the sea urchin larvae [21]. Similarly, we found that expression of *NvFoxJ1* [62] is first detectable at the midplanula stage as a spot at the aboral pole, coincident with the development of the apical organ (Figure 1E1–E7).

From these data it is evident that a subset of bilaterian head development genes and components of the FGF signalling pathway are expressed at the aboral pole of *Nematostella* with distinct dynamics. At the blastula and gastrula stages, *NvSix3/6*, *NvFoxQ2a*, *NvIrx*, *NvFGFa1*, *NvFGFa2*, and *NvFGFRa* are broadly expressed at the aboral pole, but after gastrulation they segregate into mutually exclusive domains: *NvSix3/6*, *NvFoxQ2a*, and *NvFoxD1* [63] are expressed in a broad ring surrounding the apical organ (“ring genes,” Figure 1G), whereas the expression of *NvIrx*, *NvFGFa1*, *NvFGFa2*, and *FGFRa* becomes restricted to the apical organ (“spot genes,” which include also *NvSoxB(1)* and *NvHoxF/Anthox1*, Figure 1H). *NvFoxJ1* exemplifies a third type of aboral expression pattern, with an onset of expression as a spot only after gastrulation (“late genes,” Figure 1I), probably indicating a function in the differentiation of the apical organ cells.

### FGF Signalling Is Required to Exclude *NvSix3/6* from the Apical Organ Domain

In order to understand how the observed dynamics of the expression patterns relate to the development of the aboral region, we conducted a series of morpholino (MO)-mediated gene knockdown experiments. Injection of two different control morpholinos had no effect on the development or on gene expression patterns. The expression of EGFP from specific reporter mRNAs was blocked when co-injected with the gene specific, but not with the control morpholinos (Figure S4 and Materials and Methods).

**Figure 2:**
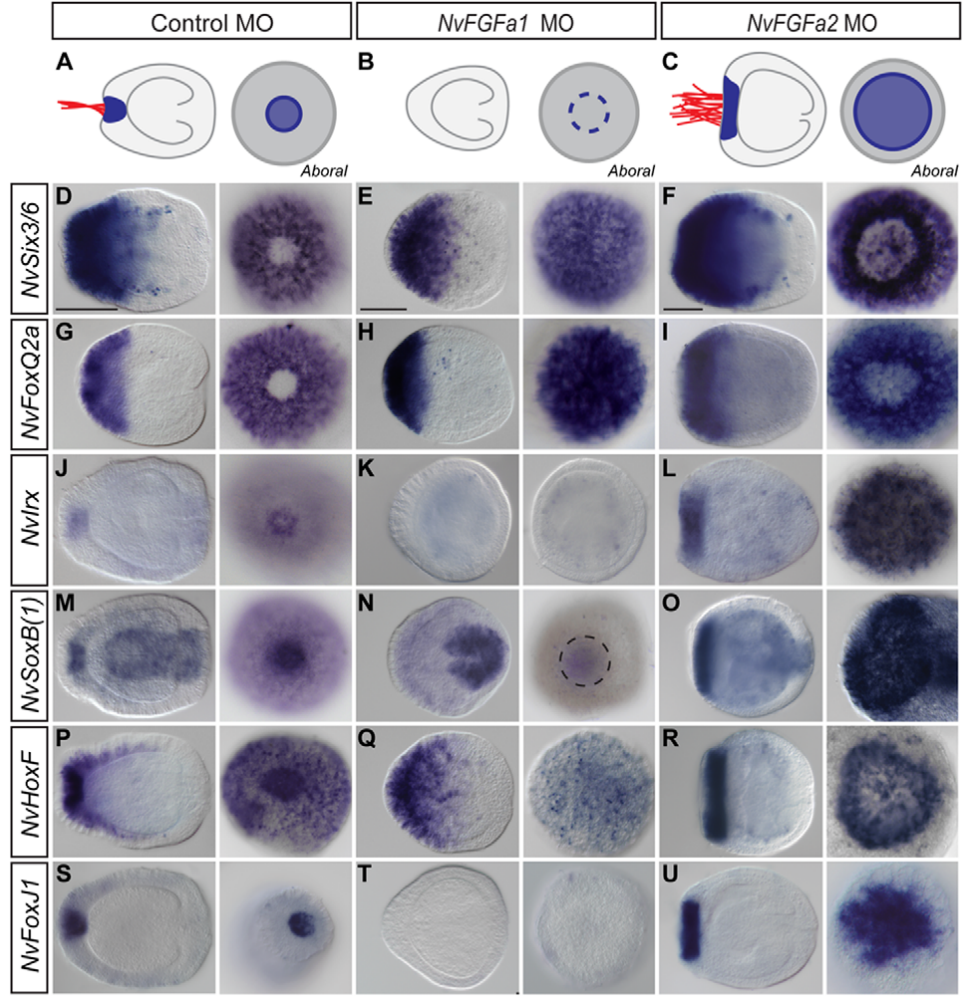
FGF signalling specifically controls gene expression in the apical organ territory. (A–C) Schematic illustration of the phenotype caused by knockdown of *NvFGFa1* (B) and *NvFGFa2* (C); *NvFGFa1* morphants lack the apical tuft (indicated in red) and the small indentation at the aboral pole, from which it develops. *NvFGFa2* morphants have an expanded indentation and apical tuft. (D–U) In situ hybridizations at the midplanula stage (72 h postfertilization, hpf). Morpholinos are indicated above the images, with in situ probes on the left side. (D–I) The aboral gap in the expression of “ring genes” is lost in *NvFGFa1* and expanded in *NvFGFa2* morphants. (J–U) Expression of “spot” and “late genes” is abolished in *NvFGFa1* morphants and expanded in *NvFGFa2* morphants. (N and Q) Note that knockdown of *NvFGFa1* specifically affects the aboral expression of *NvSoxB(1)* and *NvHoxF/Anthox1.* For each experimental condition and analysis, a lateral view with aboral pole to the left is shown next to an aboral view (lateral view, left; aboral view, right); all images at the planula stage. Scale bar, 100 µm.

We have previously shown that the development of the apical organ in *Nematostella* is controlled by the opposing activities of two FGF ligands, with *NvFGFa1* being essential for apical organ development and *NvFGFa2* counteracting FGF receptor activity to limit the size of the apical organ ([72]; Figure 2A–C). We used the morpholinos (MOs) against *NvFGFa1* and *NvFGFa2* in order to understand the dependence of the different aborally expressed genes on FGF signalling. Injection of *NvFGFa1* MO does not affect the width of the *NvSix3/6*, *NvFoxQ2a*, and *NvFoxD1* expression domains at the planula stage, however the spot-like gap at the aboral pole is not present any longer (Figure 2D, E, G, H and Figure S5). Expression of the “spot-genes” *NvIrx*, *NvSoxB(1)*, and *NvHoxF/Anthox1* and of the “late gene” *NvFoxJ1* is suppressed (Figure 2J, K, M, N, P, Q, S, T) in a specific manner, as demonstrated by unaffected expression of *NvSoxB(1)* (Figure 2N) and *NvHoxF/Anthox1* (Figure 2Q) in domains outside the aboral pole. In contrast, injection of *NvFGFa2* MO, which results in an expansion of the apical organ [72], leads to the opposite effect. For *NvSix3/6* and *NvFoxQ2a*, the gap in the expression domains at the aboral pole is expanded (Figure 2F and I), whereas for the “spot genes” and “late genes” the aboral expression domain is enlarged (Figure 2L, O, R, U). These results suggest that the broad aboral expression of *NvSix3/6* and *NvFoxQ2a* is independent of FGF signalling but that their suppression at the aboral pole after gastrulation requires FGF activity. Furthermore, the expression of “spot” and “late” genes at the planula stage requires FGF signalling, suggesting an apical organ-specific function of the FGF pathway.

### *NvSix3/6* Is Required for the Development of the Aboral Region

**Figure 3:**
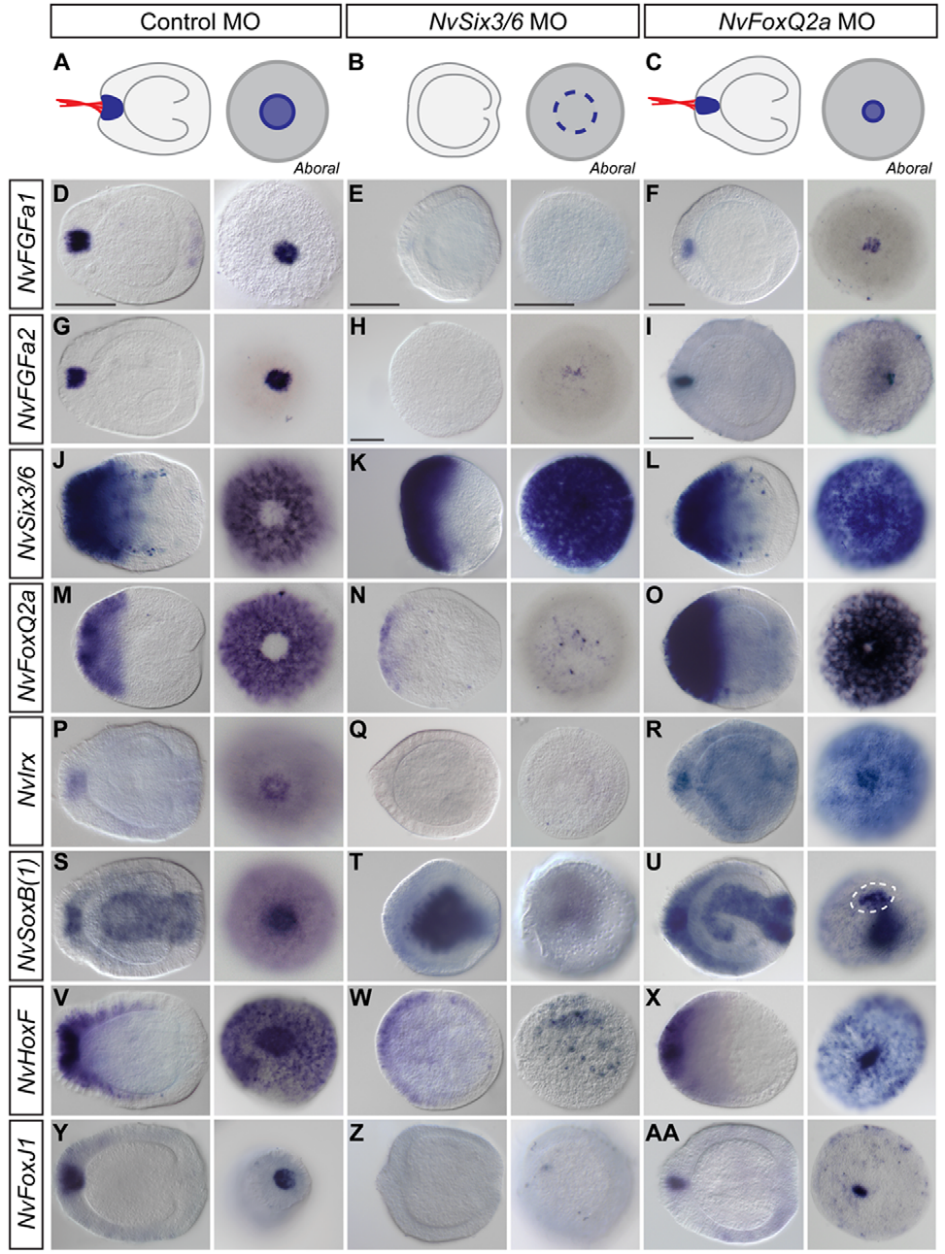
*NvSix3/6* controls the development of the aboral territory. (A–C) Illustration of the morphological phenotype after knockdown of *NvSix3/6* (B) and *NvFoxQ2a* (C). (B) *NvSix3/6* morphants have a shortened oral-aboral body axis and lack the apical tuft. (C) *NvFoxQ2a* morphants have a smaller apical tuft. (D–AA) In situ hybridizations at the midplanula stage (72 hpf); morpholinos are indicated above the images, with in situ probes on the left side. (E, H, K, N, Q, T, W, and Z) In *NvSix3/6* morphants, expression of all aboral pole markers except *NvSix3/6* is reduced. (T) Pharyngeal expression of *NvSoxB(1)* is retained and visible in the centre of the aboral view. (W) Note that *NvHoxF/Anthox1* expression in the apical organ and in scattered aboral cells is absent/reduced. (F, I, L, O, R, U, X, and AA) *NvFoxQ2a* morpholino injection leads to a modest reduction in the expression of “spot” and “late genes” and a corresponding reduction in the gap of “ring genes.” For each experimental condition and analysis, a lateral view with aboral pole to the left is shown next to an aboral view (lateral view, left; aboral view, right); all images at planula stage. Aboral view in (U) is tilted to allow discrimination of the aboral (dashed circle) from pharyngeal expression. Scale bar, 100 µm.

As the broad aboral expression of *NvSix3/6* and *NvFoxQ2a* is unaffected by the loss of FGF signalling, we tested whether these genes act upstream of FGF signalling. Injection of a translation-blocking *NvSix3/6* MO resulted in planulae that do not extend normally along the oral-aboral axis and that lack the apical tuft (Figure 3A and B). The expression of *NvFGFa1*, *NvFGFa2*, *NvFGFRa*, *NvFoxQ2a* and *NvFoxD1*, *NvFoxJ1*, and the other spot genes is absent or strongly reduced in *NvSix3/6* MO-injected planulae (Figure 3D and E, G and H, M and N, P and Q, S and T, V and W, Y and Z, and Figure S6), whereas the expression of *NvSix3/6* itself is maintained, except for the absence of the gap corresponding to the apical tuft region (Figure 3J and K). Injection of two different translation-blocking morpholinos against *NvFoxQ2a* has a comparably small effect, since the overall morphology of the planula is unaffected, and the apical organ, although smaller, is still present. In situ hybridization analysis shows slightly reduced expression of the “spot genes” (Figure 3F, I, R, U, X, AA) and a smaller gap of the “ring genes” *NvSix3/6* and of *NvFoxQ2a* itself (Figure 3L, O).

Thus, at the planula stage, *NvSix3/6* acts upstream of the other analysed genes, suggesting that it represents a key regulator for the aboral region.

### Overactivation of FGF Receptor Signalling Rescues Apical Organ Formation in *NvSix3/6* Morphants

**Figure 4:**
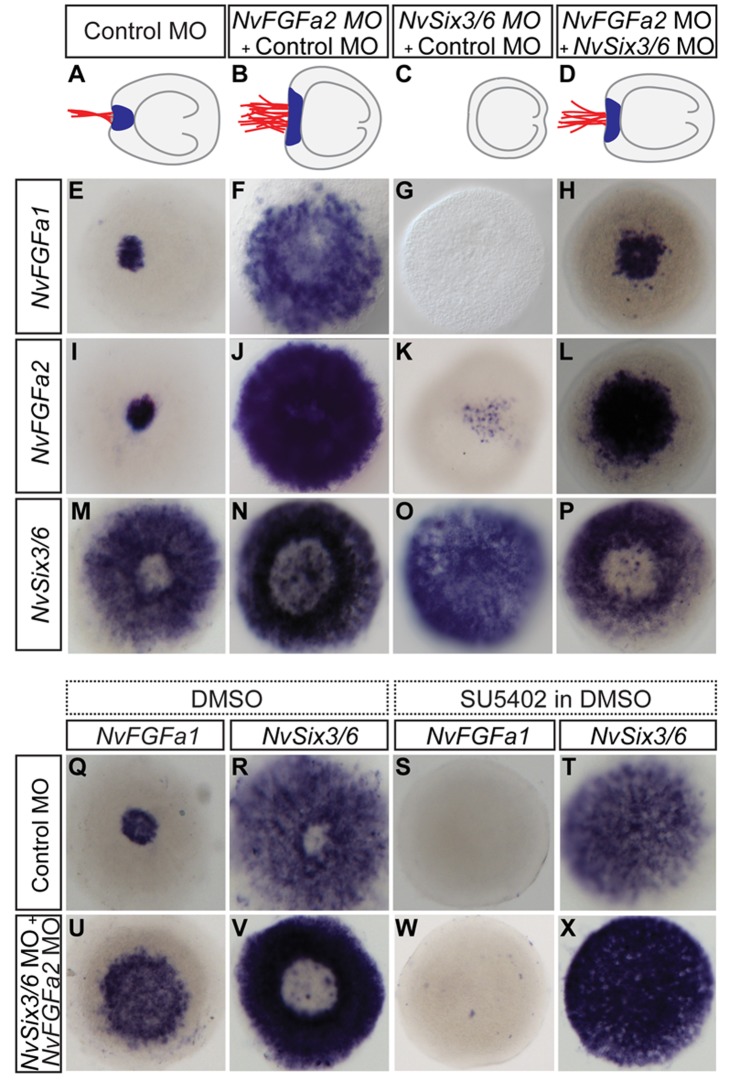
FGF receptor signalling suppresses *NvSix3/6* expression after gastrulation and is sufficient to induce apical organ formation. (A–D) Schematic representations of the morphological phenotype obtained after morpholino double injections. (D) Co-injection of *NvSix3/6* and *NvFGFa2* morpholinos reverses the loss of the apical tuft of *NvSix3/6* morphants (C). (E–P) Aboral views of in situ hybridizations at the midplanula stage (72 hpf); probes are indicated on the left side and morpholinos on top. Co-injection of *NvSix3/6* and *NvFGFa2* morpholinos leads to a moderate expansion in the expression of “spot genes” (E–L) and expansion of the aboral gap of the *NvSix3/6* expression (M–P). (Q–X) Effects of FGFR inhibitor on the double injected animals. Probes are indicated above the images, with morpholinos on the left side. Embryos were treated, from the late gastrula stage on, with DMSO (Q, R, U, V) or with the FGF receptor inhibitor SU5402 in DMSO (S, T, W, X). Inhibition of FGFR activity suppresses apical organ formation in double injected embryos. Scale bar, 100 µm.

The injections of *NvSix3/6* and *NvFGFa1* morpholinos indicated that *NvSix3/6* may act at an earlier stage than (and probably upstream of) FGF signalling in the development of the aboral pole. To test more precisely the epistatic relation between *NvSix3/6* and NvFGF signalling, we simultaneously blocked *NvSix3/6* and overactivated FGFR signalling. To achieve overactivation of FGFR signalling, we suppressed the translation of *NvFGFa2*, since we had previously shown that the expansion of the apical organ after *NvFGFa2* MO injection is caused by excessive FGFR signalling [72]. While injection of *NvSix3/6* MO leads to a loss and *NvFGFa2* MO to an expansion of the apical organ and corresponding changes in marker gene expression (Figure 4A–C, E–G, I–K, M–O), co-injection of *NvSix3/6* MO and *NvFGFa2* MO restores the development of the apical organ, resulting in a moderately expanded structure (Figure 4D, H, L, P).

To confirm that this effect is indeed caused by FGFR activation, we treated the *NvSix3/6* MO + *NvFGFa2* MO injected animals with the FGFR inhibitor SU5402 in DMSO [72],[75] or with only DMSO as a control. In order to avoid defects in gastrulation, we started the treatment at the end of this phase. SU5402 treatment of the double-injected animals results in planulae lacking the apical organ, and in situ hybridization shows suppression of aboral *NvFGFa1* expression and a lack of the gap in the *NvSix3/6* expression (Figure 4Q–X).

Thus, activation of FGF receptor signalling is necessary and sufficient for apical organ formation even when translation of *NvSix3/6* is blocked, suggesting that *NvSix3/6* is acting early in aboral domain development, upstream of FGFR signalling. Furthermore, since the SU5402 treatment was started after gastrulation, FGFR signalling appears to have a distinct function at a late stage of aboral domain development.

### *NvHoxF/Anthox1* and *NvSoxB(1)* Are Required for Apical Organ Formation

**Figure 5:**
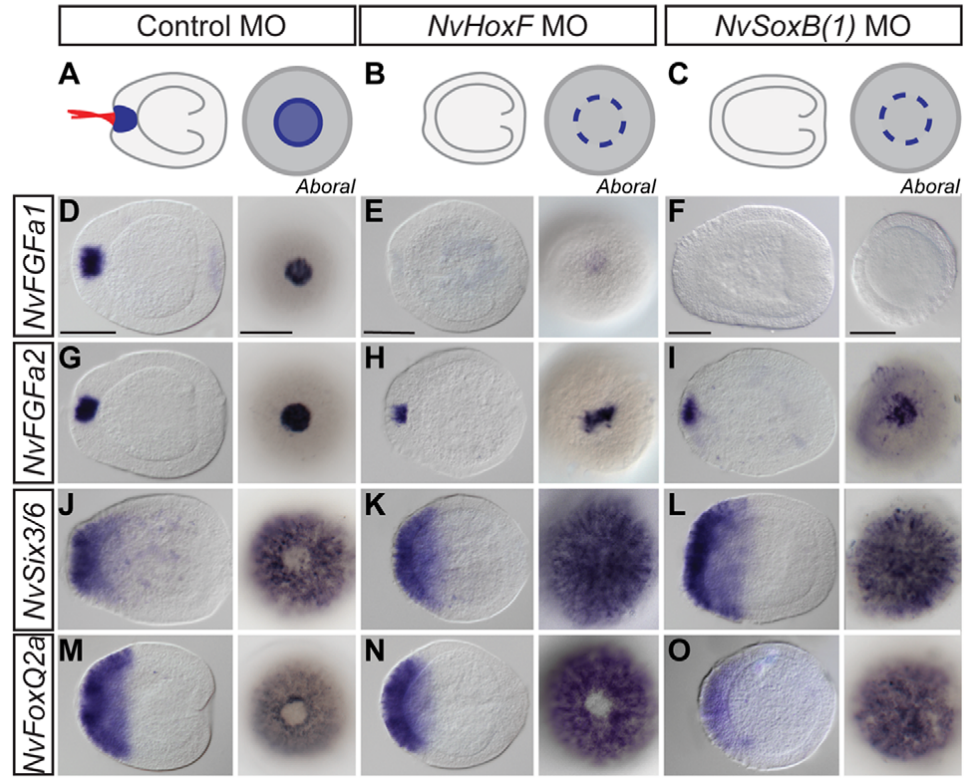
*NvHoxF/Anthox1* and *NvSoxB(1)* have distinct roles in the regulation of aboral domain and apical organ genes. (A–C) Schematic drawings illustrating the morphology of *NvHoxF/Anthox1* (B) and *NvSoxB(1)* MO-injected planulae. Both genes are required for apical tuft formation, but only *NvSoxB(1)* morphants show a slightly elongated body column (C). (D–O) In situ hybridizations, lateral views (left) with the aboral pole to the left are shown next to aboral views (right) at the midplanula stage (72 hpf). In situ probes are indicated on the left, with morpholinos on top. *NvHoxF/Anthox1* and *NvSoxB(1)* are required for the expression *of NvFGFa1* (D–F) but not *NvFGFa2* (G–I). *NvSoxB(1)* morphants lack the gap in the expression of *NvSix3/6* and *NvFoxQ2a* (L, O), while *NvHoxF/Anthox1* morphants only lack the gap in *NvSix3/6* expression (K, N). Scale bar, 100 µm.

We have shown above that the apical organ expression of *NvHoxF/Anthox1* and *NvSoxB(1)* is absent in *NvFGFa1* morpholino-injected animals. Moreover, at the planula stage, *NvFGFa1* is required for the expression of *NvFGFa2* and *NvFGFa1* itself [72]. Surprisingly, we found that morpholinos targeting *NvHoxF/Anthox1* or *NvSoxB(1)* have differential effects on the expression of the two FGFs. While injection of *NvHoxF/Anthox1* MO and *NvSoxB(1)* MO both result in planulae lacking the apical tuft (Figure 5A–C), only the expression of *NvFGFa1* becomes undetectable (Figure 5D–F), whereas the expression of *NvFGFa2* is slightly reduced (Figure 5G–I). Likewise, in *NvHoxF/Anthox1* MO-injected planulae, only the gap in the expression of *NvSix3/6* but not of *NvFoxQ2a* is absent (Figure 5J, K, M, N), while *NvSoxB(1)* MO leads to a loss of the gap in both *NvSix3/6* and *NvFoxQ2a* expression domains (Figure 5L, O).

These results show that, although *NvFGFa1* is required for the expression of *NvFGFa1* and *NvFGFa2* and for the suppression of *NvSix3/6* and *NvFoxQ2a* in the apical organ, the regulation of these genes involves at least partially different regulatory inputs. Whether these differences are of quantitative or qualitative nature remains to be determined.

### Regulatory Interactions Change between the Gastrula and Planula Stages

**Figure 6:**
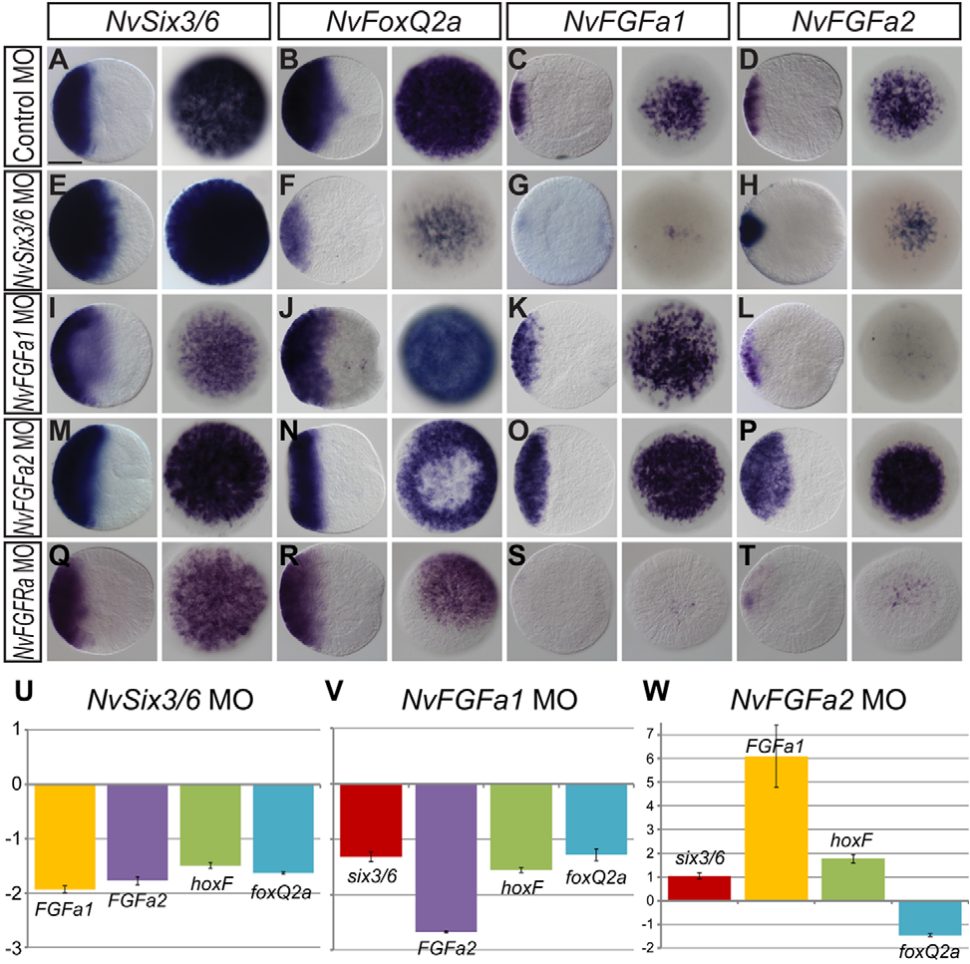
*NvSix3/6* and *NvFGFRa* are required for the initiation of a FGF signalling feedback loop at gastrulation. (A–T) In situ hybridizations with probes indicated on top and injected morpholinos on the left. All animals are at the midgastrula stage (24 hpf); lateral views with aboral pole to the left are shown next to aboral views. In *NvSix3/6* morphants, the expression of *NvFGFa1* and *NvFGFa2* is reduced (C, D, G, H). In contrast to the situation at the planula stage, the expression of *NvFGFa2* but not *NvFGFa1* is reduced in *NvFGFa1* morphants (K, L). *NvFGFRa* is required for the expression of *NvFGFa1* and *NvFGFa2*, but not for *NvSix3/6* and *NvFoxQ2a* (Q–T). (U–W) Quantitative RT-PCR of (U) *NvSix3/6* MO-, (V) *NvFGFa1* MO-, and (W) *NvFGFa2* MO-injected embryos at the midgastrula stage (24 hpf). Fold changes of the relative expression levels of the indicated genes are shown; values between [−1, +1] mean no change, and +2 corresponds to 100% increase. Error bars represent the standard deviation of three biological replicates.

Until the midgastrula stage, “ring” and “spot” genes share a broad aboral expression domain that only at the late gastrula stage starts to segregate into mutually exclusive domains (Figure 1). This suggests that the regulatory interactions between these genes may change after gastrulation. To test this scenario, we analysed the effect of morpholino injections at the midgastrula stage by in situ hybridization to detect potential spatial changes in expression and by quantitative RT-PCR to detect changes in expression levels that may not affect the extension of the expression domains. Since morpholinos can affect the stability of the targeted mRNA, we excluded the respective target genes from the qPCR analysis. Consistent with the observed effect at the planula stage, injection of *NvSix3/6* morpholino has no negative effect on the expression of *NvSix3/6* itself (Figure 6A, E), but substantially reduces the expression of *NvFoxQ2a*, *NvFGFa1*, and *NvFGFa2* (Figure 6B–D, F–H, U).

Injection of *NvFGFa1* morpholino reveals differential regulation at the gastrula and planula stages, respectively. In contrast to the planula stage, the expression of *NvFGFa1* itself is rather up-regulated at the gastrula stage (Figure 6K), whereas *NvFGFa2* is downregulated (Figure 6L, V), as it is at the planula stage. Interestingly, injection of a morpholino-targeting *NvFGFRa* strongly reduces the expression of *NvFGFa1* and *NvFGFa2* at the gastrula stage (Figure 6S, T), while it has no effect on the expression of *NvSix3/6* and *NvFoxQ2a* (Figure 6Q, R). This suggests that an as yet unidentified FGF is required for the early expression of *NvFGFa1* and *NvFGFa2*. This idea is supported by the increase in expression of these two genes upon knockdown of the inhibitory *NvFGFa2* (Figure 6O, P, W).

Thus, the main difference in the interactions of these genes in the gastrula stage compared to the planula stage is a lack of positive feedback of *NvFGFa1* on its own expression at the gastrula stage, suggesting that the autoregulatory FGF signalling loop becomes fully established only after gastrulation.

### The Aboral Domain Acquires a More Central Fate in *NvSix3/6-*Depleted Planulae

**Figure 7:**
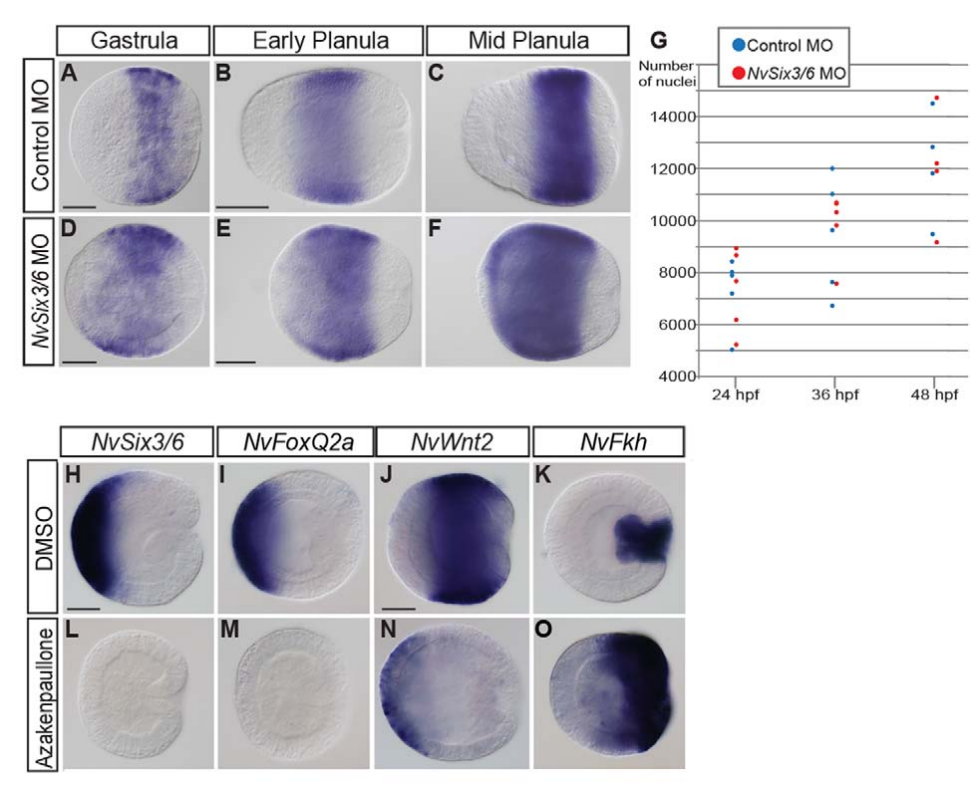
*NvSix3/6* and canonical Wnt signalling interact in aboral domain development. (A–F) Lateral views with aboral pole to the left of in situ hybridizations with a *NvWnt2* probe from gastrula stage to the mid-planula (stages indicated on top), injected with a control (A–C) or an *NvSix3/6* (D–F) morpholino. The expression domain of *NvWnt2* progressively expands toward the aboral pole upon knockdown of *NvSix3/6*. (G) Quantification of the number of DAPI stained nuclei in control MO- (blue) and *NvSix3/6* MO- (red) injected embryos. Number of nuclei is indicated on the *y*-axis, with time point of analysis on the *x*-axis. (H–O) Lateral views at the gastrula stage, with aboral pole to the left. Probes are indicated on the top, with treatments on the left. At 2 µM Azakenpaullone, expression of aboral markers is suppressed, and central and oral markers are shifted and expanded aborally, respectively. Scale bar, 100 µm.

The analysis of *NvSix3/6* MO-injected animals has shown that *NvSix3/6* is required for the proper expression of all aboral marker genes except for *NvSix3/6* itself, suggesting that *NvSix3/6* is acting at a high level in the regulatory network that controls the specification of the aboral domain. To support this hypothesis we studied the expression of a marker gene for the central domain of the *Nematostella* planula, *NvWnt2*. *NvWnt2* is expressed in a belt-like domain midway between the oral and aboral poles (Figure 7A–C; [49]), and it is essentially unaffected by loss of NvFGFRa signalling [72].

Strikingly, in *NvSix3/6* MO-injected animals, the expression of *NvWnt2* extends progressively to the aboral pole, while the oral expression boundary is unaffected (Figure 7D–F). This is consistent with the hypothesis that at least part of the aboral domain acquires a more central fate. However, *NvSix3/6* MO-injected planulae appear shortened compared to control planulae, which could indicate that instead of being respecified, the cells of the aboral domain are not generated at all or undergo apoptosis [76],[77]. To distinguish between these possibilities, we quantified the number of nuclei at different time points. We could not detect a significant difference in the number of nuclei in *NvSix3/6* MO versus control planulae (Figure 7G).

This suggests that the observed aboral expansion of the *NvWnt2* expression domain in *NvSix3/6* MO-injected animals reflects a respecification of the aboral domain to a more oral fate.

Mutual repression of *six3* and canonical Wnt signalling has been observed in vertebrates and sea urchins [11],[20],[78],[79], and the aboral expression of *foxQ2* in the hydrozoan *Clytia* expands orally when *Wnt3* is inactivated [51]. To test whether a similar situation is present during *Nematostella* development, we overactivated canonical Wnt signalling by chemically inhibiting Glycogen Synthase Kinase 3 (GSK3), a negative regulator of the Wnt pathway. Incubation with 2 µM of the GSK3 inhibitor Azakenpaullone [80] leads to a complete suppression of aboral *NvSix3/6* and *NvFoxQ2a* expression at gastrulation (Figure 7H, I, L, M) and a shift of the expression of *NvWnt2* and the oral marker *NvFkh* [81],[82] towards the aboral pole (Figure 7J, K, N, O). At higher concentrations (5 µM), *NvWnt2* expression is lost and *NvFkh* is expressed throughout the embryos (L.L. and F.R., unpublished observation). Thus, similar to some bilaterians, *NvSix3/6* is required to prevent ectopic *wnt* expression and can itself be repressed by ectopic canonical Wnt activity.

### *NvSix3/6* Is Dispensable for Neurogenesis But Required for Morphogenesis and Cell-Type Specification in the Aboral Area

**Figure 8:**
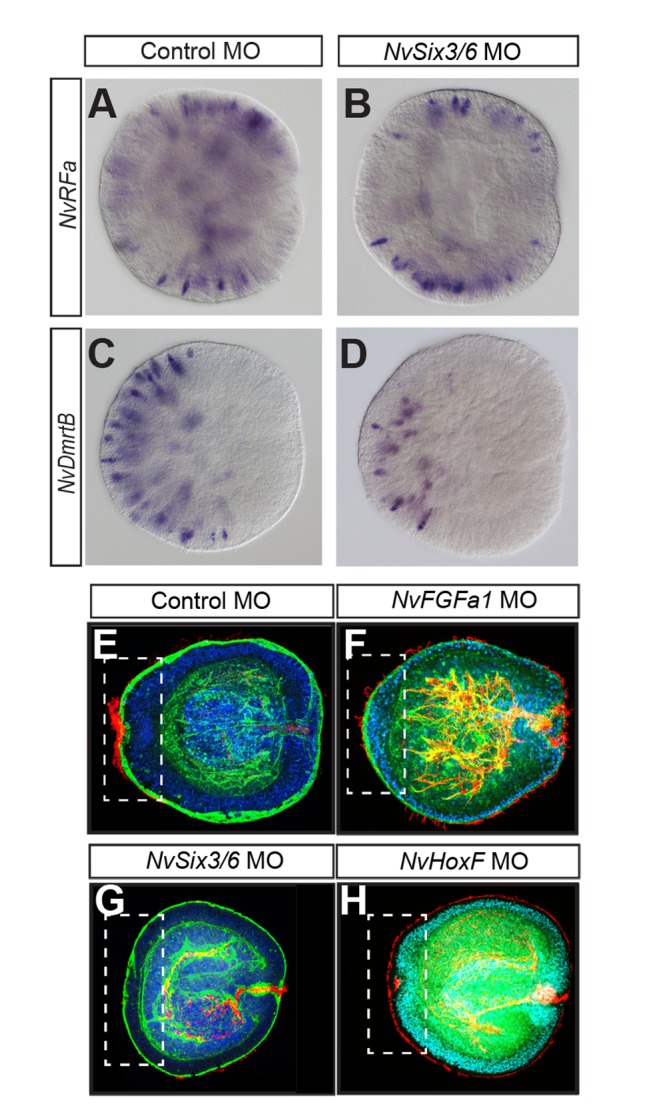
Reduction of aboral neural markers and loss of aboral morphology in *NvSix3/6* morphants. (A–D) Lateral views of in situ hybridizations at the gastrula stage, with aboral pole to the left. Probes are indicated on the left, with morpholinos on top. Injection of *NvSix3/6* MO leads to a reduction of aboral *NvDmrtB* positive neurons, but not to a general loss of neurons. (E–H) Lateral views of midplanula (72 hpf) animals labelled with DAPI (blue), Phalloidin (green), and anti-acetylated tubulin antibody (red). Aboral pole is to the left, and injected morpholinos are indicated on top. Dashed boxes mark the apical organ region; *NvFGFa1* morphants lack basally positioned nuclei (F); in *NvSix3/6* morphants (G), no difference between aboral and central ectoderm is visible. In *NvHoxF/Anthox1* morphants, the aboral indentation with basal migration of aboral pole nuclei occurs, but no apical tuft develops (H).

In vertebrates, sea urchin, and *Tribolium*, suppression of *six3* function impairs neural development [11],[19],[20],[77]. To test whether this is also the case in *Nematostella*, we used two neural markers. *NvRFa* encodes a neuropeptide and is expressed in individual cells along the entire oral-aboral axis at the gastrula stage (Figure 8A and [83]). Injection of *NvSix3/6* MO does not have a significant effect on the *NvRFa* expression (Figure 8B). *NvDmrtB* is a transcription factor that is expressed in individual cells in the aboral half of the gastrula and that is involved in neural development (Figure 8C; [84]). Individual cells expressing *NvDmrtB* are still present at the aboral pole of *NvSix3/6* MO-injected gastrulae, but their number is reduced and they are restricted to a smaller aboral territory (Figure 8D). These observations indicate that *NvSix3/6* is not required for neurogenesis in general, but is involved in the specification of aboral neural cell types, presumably as a consequence of a more general role in aboral development.

Planulae injected with *NvFGFa1* or *NvSix3/6* morpholinos both lack an apical organ, but their overall morphology is strikingly different. To better understand the roles of these two genes, we stained injected animals with the nuclear stain DAPI and the F-actin stain Phalloidin to visualize their tissue morphology. In control MO-injected planulae, the ectodermal cells in the aboral region acquire a columnar morphology after gastrulation, whereas ectoderm cells in more central areas have a cuboidal morphology (Figure 8E). The nuclei of the cells in the centre of the aboral region migrate to a more basal position before these cells form the long cilia of the apical tuft (Figure 8E; [85]). In *NvFGFa1* MO-injected planulae, the ectodermal cells in the aboral region still become columnar, but the basal migration of nuclei does not occur (Figure 8F). In contrast, in *NvSix3/6* MO-injected planulae, the cells of the aboral region retain a cuboidal morphology and nuclei do not migrate to a more basal position (Figure 8G). Thus, consistent with the effect on the expression of *NvWnt2*, the aboral ectoderm of planulae injected with *NvSix3/6* MO displays a morphology resembling that of more central ectoderm, while *NvFGFa1* morphants specifically fail to initiate apical organ formation at the centre of the aboral region. Interestingly, in planulae injected with the *NvHoxF/Anthox1* morpholino, basal migration of aboral nuclei still occurs, but the outgrowth of long cilia is blocked, suggesting that *NvHoxF/Anthox1* acts at a late stage of apical organ formation (Figure 8H).

The broader requirement of *NvSix3/6* for the development of the aboral pole is also reflected in the expression of *NvHoxF/Anthox1*. In addition to the aboral pole expression, *NvHoxF/Anthox1* is expressed in scattered cells that are enriched in the aboral half of *Nematostella* planulae (Figure 2P). In *NvSix3/6* morphants, the apical organ expression is absent and the expression in individual cells is strongly reduced (Figure 3W). In contrast, in *NvFGFa1* morphants, only the apical organ expression is absent, but expression in the scattered cells persists (Figure 2Q).

Taken together, *NvSix3/6* is required for the development of the whole aboral territory, whereas *NvFGFa1* has a specific function in the development of the apical organ.

## Discussion

### Control of Aboral Domain Development in *Nematostella* by *NvSix3/6* and FGF Signalling

Our functional analyses lead us to a two-step model in which *NvSix3/6* acts as a key regulator of the development of the aboral domain of *Nematostella vectensis*. First, the loss of *NvFGFa1* and *NvFoxQ2a* expression in *NvSix3/6* morphants already at the gastrula stage suggests that *NvSix3/6* is required during early stages to determine the identity of the whole aboral region. In a second phase, after gastrulation, NvFGFa1 signalling via NvFGFRa is required to suppress the expression of *NvSix3/6*, *NvFoxQ2a*, and *NvFoxD1* at the aboral pole. This local suppression of the “ring genes” is necessary to allow the differentiation of the apical organ cells, with the activation of a specific cassette of genes that includes the presumably ciliary gene *NvFoxJ1*.

**Figure 9:**
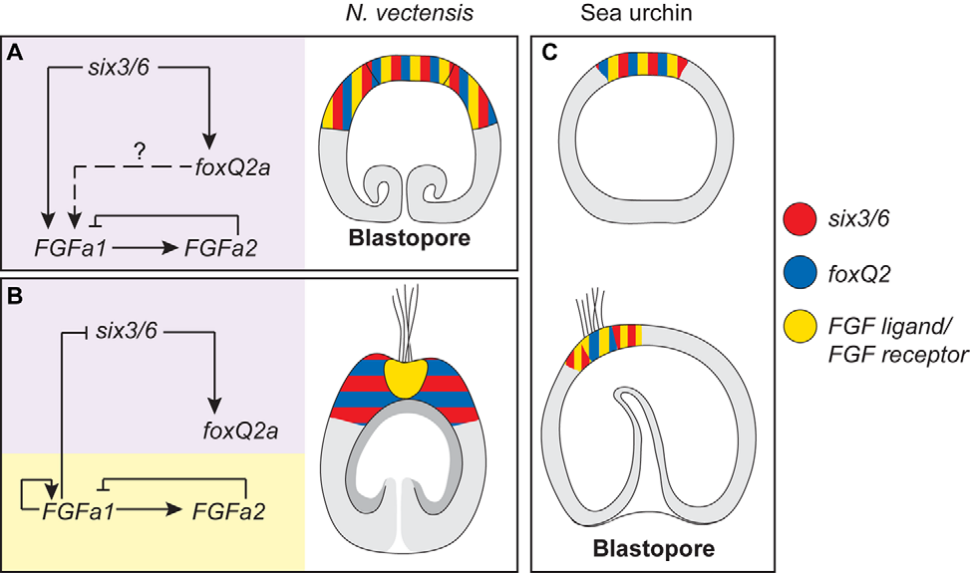
A conserved anterior patterning system in *Nematostella* planula and sea urchin larva. (A and B) Model for gene regulatory network in place at gastrula and planula stages during aboral patterning of *Nematostella* larvae. At the gastrula stage (A), all the genes are co-expressed in the aboral area and *NvSix3/6* positively regulates the expression of FGF ligands and *NvFoxQ2a*. The positive regulation of *NvFGFa2* by *NvFGFa1* is already in place, and it is likely responsible for the early restriction of *FGFs*' expression domains. At the planula stage (B), the aboral territory is divided in two domains, one surrounding the apical organ (in purple and blue/red) and one in the apical organ (orange and yellow). The first corresponds to the expression domain of *NvSix3/6* and *NvFoxQ2a* (and *NvFoxD1*), the latter to the expression of FGF ligands (drawing). The regulatory interactions change at this stage and *NvFGFa1* represses *NvSix3/6*; additionally, *NvFGFa1* starts a positive autoregulation (direct or indirect), responsible for the maintenance of the *FGF* expressing area. (C) Segregation of expression domains in sea urchin larva. Similar to *Nematostell*a gastrula, the genes are co-expressed at the contra-blastoporal (anterior) pole of sea urchin (data from *Strongylocentrotus purpuratus* and *Paracentrotus lividus*) at early blastula and then segregate in distinct domains from the mesenchymal blastula stage on, when the tuft of cilia appears. In this case, however, the genes segregate in a slightly different way: *six3* forms a ring around the apical plate, while *foxQ2* is restricted to the apical plate itself. The apical domain expression of the FGF receptor is restricted to the apical plate.

Regarding the development of the apical organ, the key function of *NvSix3/6* appears to be the initiation of an autoregulatory loop involving *NvFGFa1* and *NvFGFa2*, which uses positive (auto-activation of *NvFGFa1*) and negative (activation of the inhibitory *NvFGFa2*) feedback. This system is probably autonomously able to control the expression of FGF ligands and restrict their expression to the most aboral part of the larva, where the apical organ will form, after the down-regulation of *NvSix3/6* itself (Figure 9A,B). Thus, although inhibition of *NvSix3/6* or *NvFGFa1* both leads to a lack of the apical organ, this phenotype reflects two different developmental roles: *NvSix3/6* has a broad and early role in determining the identity of the aboral region, while *NvFGFa1* has a later, apical organ-specific function. However, in contrast to knockdown of *NvFGFa1*, morpholinos targeting *NvFGFRa* suppress the expression of *NvFGFa1* and *NvFGFa2* already at the gastrula stage, suggesting that an as yet unidentified *NvFGF* ligand might be involved in the initiation of the *NvFGFa1*–*NvFGFa2* feedback loop. The *Nematostella* genome contains 15 *FGF*s, of which 10 have not been characterized yet [71],[72] and may play a role in this process.

### Conserved Regulation of Anterior Development in Bilateria and Aboral Development in Cnidaria

*Six3*, *foxQ2*, and *FGF* genes have been shown to be expressed at the anterior/apical pole of protostome and deuterostome embryos and larvae (summarized for sea urchin in Figure 9; see also Figure S1 and [9],[10],[15],[17],[18],[21],[22],[86]–[89]), suggesting that these genes are part of a conserved regulatory module for anterior development. The role of *six3* has been analysed in detail in the sea urchin *Strongylocentrotus purpuratus* [11]. As in *Nematostella*, sea urchin *six3* is a key regulator of the apical domain—that is, the domain opposite to the gastrulation site. The sea urchin apical pole domain is characterized by an initial thickening of the ectodermal epithelium, which subsequently gives rise to the long cilia-bearing cells of the apical organ, and various surrounding cells, including serotonergic neurons (reviewed in [90]). In embryos injected with a *six3* morpholino, the ectodermal thickening does not occur and neither the apical organ nor the neurons develop [11]. Microarray analysis revealed that in sea urchin *six3* is a positive regulator of the expression of *foxQ2*, *foxJ1*, and *frizzled 5/8* [11], genes that are also expressed at the aboral pole of *Nematostella* downstream of *NvSix3/6* (Figures 1 and 3; [55] and unpublished data).

However, despite the well-conserved overlapping expression in anterior/apical domains, there are also differences between the *Nematostella* and bilaterian expression domains. For example, while the expression patterns of *NvSix3/6* and *NvFoxQ2a* are indistinguishable throughout development, their expression in sea urchin is highly similar only until the midblastula stage, when *six3* expression becomes excluded from the centre of the apical plate, while *foxQ2* remains expressed in this central apical domain of the sea urchin embryo (Figure 8B,C). Similarly, the anterior expression domain of *six3* in the hemichordate *Saccoglossus kowalewskii* and the brachiopod *Terebratalia transversa* is slightly wider than that of *foxQ2* [9],[18],[87]. This suggests that although *six3* and *foxQ2* have an ancient function in anterior development, there have been modifications in their exact interactions, indicating that even very ancient developmental modules retain some degree of plasticity.

### The Relation of the Cnidarian Oral-Aboral Axis to the Anterior-Posterior Axis of Bilaterians

Integration of the data presented here with studies in other cnidarians and in bilaterians shows that the aboral domain of cnidarians and the head-forming anterior domain of bilaterians share the expression of several transcription factors and signalling molecules (Figures 9 and S1). This suggests that in the last common ancestor of cnidarians and bilaterians, aboral (i.e., contrablastoporal) development was controlled by *six3/6* and the aboral domain was additionally characterized by the expression of *foxQ2*, *frizzled 5/8*, *rx*, *irx*, and probably *FGF* signalling genes and *foxD1*. The most parsimonious explanation of these observations is that the anterior part and thus the head of bilaterians is derived from the aboral domain of the cnidarian-bilaterian ancestor.

The alternative hypothesis that cnidarians do not have a region homologous to the bilaterian head is based on the inconsistent expression of orthologs of some bilaterian anterior markers in cnidarians. However, the notion of a conserved aboral/anterior pattern mechanism does not require that all bilaterian anterior genes are already involved in cnidarian aboral development. Rather, we conjecture that additional genes became integrated into a more elaborate bilaterian anterior patterning system. This is likely for some genes that do not have clear orthologs in cnidarians (e.g., *pax6* or *nk2.1*). It could also be the case for *orthodenticle/otx* genes whose ancestral function might have been in endoderm development [43],[58],[91]–[96] or for *orthopedia/otp* genes, which mark restricted anterior neural domains and cell types (e.g., dopaminergic neurons) in bilaterians [9],[18],[97]–[99] and which consistent with the lack of a brain is not aborally expressed in *Nematostella* [100]. Analysis of additional bilaterian anterior markers (e.g., *fez*, *foxG1*, *tailless*) in cnidarians will help to define a putative ancient aboral/anterior patterning system.

Our data are not consistent with the hypothesis in which the molecular determinants of the gastrulation site and those for bilaterian anterior neural development both localize to the animal pole in cnidarians and became spatially decoupled only after the site of β-catenin nuclearization moved to the vegetal pole in the ancestor of bilaterians [3]. This hypothesis predicts that conserved bilaterian anterior development genes should be expressed, and function on the oral side of cnidarians, which is not consistent with the data presented here. Instead, conserved blastoporal and anterior patterning genes localise to and function at opposite poles in cnidarians, although with reversed orientation when related to the animal-vegetal axis of the oocyte.

Evidence from several cnidarian and many bilaterian species suggests that the gastrulation site of the cnidarian-bilaterian ancestor was characterized by the activity of Wnt/β-catenin signalling. Thus, the development of the two poles of cnidarian gastrulae appears to be regulated by two conserved molecular systems. Furthermore, our data suggest that antagonism between *six3* genes and canonical Wnt signalling in axial patterning may have an early evolutionary origin. This is indirectly supported by the aboral expression of *foxQ2a* in the hydrozoan *Clytia hemispherica* [24], which is negatively regulated by orally expressed *wnt3* [51]. Interestingly, aboral expression of *foxQ2a* commences at early gastrulation in *Clytia* and at the blastula stage in *Nematostella* and therefore is a zygotic event. In contrast, *Wnt3* acts as a maternally localized factor in *Clytia*, but localized expression of *Nematostella Wnts* becomes visible only zygotically [24],[49],[51],[52].

It is worth noting that the suggested homology of the domains at the two poles of the cnidarian and bilaterian primary body axes does not necessarily mean that the complete patterning of their axes is homologous. For example, it remains to be shown that a region corresponding to the trunk region of bilaterians exists in cnidarians. Similarly, the conservation of regulators for the specification of the aboral region does not necessarily mean that the apical organs of anthozoans and bilaterians are homologues. Further studies addressing the development and function of the different cell types constituting the apical organ are necessary to resolve this question.

### Early Evolution of Head Development

The head of most bilaterians is characterized by a centralization of the nervous system, the brain. In contrast, despite regional differences in neuron density, neither *Nematostella* nor other cnidarian planulae or polyps display a comparable brain-like centralization of the nervous system at the oral or aboral end [83],[85],[101],[102]. In sea urchin and *Tribolium*, *six3* is required for anterior neural and epidermal development [11],[19], whereas in vertebrates the expression and function of *six3* is restricted to the anterior nervous system [20],[77]. Our data indicate that *NvSix3/6* is not required for neurogenesis per se, although it influences the development of the aboral *NvDmrtB* positive neurons. We cannot rule out that *NvSix3/6* has an additional, direct function in the specification of aboral neurons, but based on the effects on general aboral markers and morphology, we favour the idea that the effect on neural specification is a secondary consequence of the broader patterning function of *NvSix3/6*. In any case, the function of *six3* in anterior/aboral patterning predates the evolution of any anterior nervous system centralization and the role of anterior patterning genes may have become restricted to the nervous system in the chordate lineage. Similarly, patterning systems controlling the development of secondary brain signalling centres in vertebrates may have evolved earlier than the corresponding structures [86]. This suggests that patterning systems with tissue-specific functions (e.g., in the nervous system) may have originated as more general axial patterning modules and only subsequently acquired tissue specificity.

In conclusion, we have shown that orthologs of the bilaterian anterior patterning genes *six3*, *foxQ2*, and *irx* are expressed at the aboral pole of a cnidarian and that *NvSix3/6* is a key regulator of *Nematostella* aboral pole development. Despite the lack of a morphological correlate of the bilaterian head in cnidarians, these data provide support for the homology of the cnidarian aboral and bilaterian anterior pole and suggest that a basic head development system evolved prior to the bilaterian brain.

## Materials and Methods

### *Nematostella* Culture

The animals were cultured in the Sars Centre facility and induced for spawning as described previously [59],[103]. The embryos were grown at 21°C, in one-third filtered seawater. At this temperature, after 24 h the animals are at a midgastrula stage and after 3 d at a midplanula stage.

### Identification of the Sequences

Gene models for *NvSix3/6*, *NvIrx*, and *NvFoxJ1* were identified by Larroux et al. [62]. *NvFoxQ2a* was first identified by Magie et al. [63] as *NvFox2* and then assigned to the Q2 family by Chevalier et al. [24]; here, following the nomenclature of Larroux et al. [62] we named the gene *NvFoxQ2a*.

The complete sequences were obtained by RACE, with the SMART RACE cDNA Amplification Kit (BD Biosciences). The primer sequences are included in the Table S1. NCBI accession numbers are KC137590 (*NvSix3/6*), KC137591 (*NvFoxQ2a*), KC137592 (*NvIrx*), and KC137593 (*NvFoxJ1*).

### In Situ Hybridization and Immunostaining

In situ hybridization was performed as described previously [72],[104]. Probes were synthesized from full-length cDNA clones with Megascript Kits (Ambion), using either Digoxigenin or FITC-labeled UTP (Roche).

The immunostainings were performed as in [85], DAPI (1∶1,000 Molecular Probes) was used as nuclear stain, Alexa Fluor 488-conjugated phalloidin (1∶25, Molecular Probes) for filamentous actin, and anti-acetylated tubulin antibody (mouse, 1∶500, Sigma T6793) to label cilia.

### Morpholino Injection

Microinjections were conducted with a FemtoJet (Eppendorf), as previously described [72]. Morpholinos (GeneTools) were tested for appropriate working concentrations and then injected at a concentration of 333 or 666 nM, together with Alexa dye-coupled dextran (final concentration 50 ng/µl). As control we used two morpholinos that did not produce any hit in the *Nematostella* genome database (Joint Genome Institute): a *NvSix3/6* mismatch MO and a generic control MO, used previously. All used morpholinos are translation blocking; the sequences are presented in Table S2. The described phenotypes were observed in 60%–90% of the injected embryos in at least three independent experiments. For control experiments, the morpholino target sequences were added upstream of EGFP by PCR (added nucleotides relative to the start codon: *NvSix3/6* 5′UTR, −38 to −1; *NvFoxQ2a*, +1 to +25; *NvSoxB1*, +1 to +25; *NvHoxF*, −15 to +10), and the fragments were cloned into vector pCS2+. Capped mRNA was synthesized in vitro using the sp6 mMessageMachine kit (Ambion) and purified with NucAway spin columns (Ambion). The mRNAs (60 ng/µl) were injected together with the corresponding gene-specific and control morpholinos, respectively. EGFP mRNA without any of the morpholino target sites was used as an additional control in co-injections. Images were acquired at 24 hpf with identical settings.

### Inhibitor Treatment

The FGFR inhibitor SU5402 (Calbiochem) was applied at a final concentration of 20 µM in 0.1% DMSO, and the GSK3 inhibitor Azakenpaullone (Sigma) was applied at final concentrations of 2 µM and 5 µM in 0.1% DMSO. Control animals were incubated in 0.1% DMSO only. Solutions were applied after gastrulation and changed after 8 hours for SU5402 and from 4 h after fertilization (8–16 cell embryos) until gastrula stage for Azakenpaullone.

### qPCR

RNA of injected embryos was extracted with the RNAqueous kit (Ambion) and genomic contamination removed with TURBO DNA-free kit (Ambion). The RNA quality was analyzed with an Agilent 2100 Bioanalyzer, and the cDNA was synthesized with Super Script III RT, using random hexamers as primers (Invitrogen). The qPCR analysis was performed with a SYBR Green I kit (QIAGEN), on a CFX96 Real-time cycler (BioRad).

The efficiency of all primers was determined with tenfold dilution series: all primer pairs had efficiencies ranging between 94% and 102%. Each qPCR sample was repeated in a double technical replicate, and each analysis repeated with at least 3 biological replicates (independent injections). We tested our control genes for stability using the online tool available at the Cotton EST Database (http://www.leonxie.com/referencegene.php), and we selected *ATP synthase* (GeneID: 5511629), *EF1b* (GeneID: 5505225), and *Ribosomal Protein L23* (GeneID: 5516837). Primer sequences are provided in Table S3.

The relative changes in expression between control-injected and MO-injected were calculated with the ΔΔCt method, assuming primer efficiency of 100%. In every analysis we used at least two reference genes, whose values were then averaged for normalization. The obtained ratios are displayed as fold change: the down-regulated genes (ratio<1) are represented with the negative reciprocal value.

### Imaging

In situ pictures were taken using a Nikon Eclipse E800 and a Nikon AZ100M microscope; the images were adjusted in Photoshop CS5. The confocal images were recorded using a Leica SP5 confocal microscope; confocal stacks were processed with the Leica software and then adjusted in Photoshop. Counting of nuclei (stained with DAPI) was performed with the Imaris software (BitPlane), using the Spot algorithm.

## Supporting Information

Figure S1 Summary of the expression of bilaterian anterior genes and their cnidarian orthologs. The color code for expression categories during embryonic and larval stages is shown at the bottom, with references at the top right of each circle. The list of references can be found in Text S1. Nd, not determined; na, not applicable (no clear ortholog present in genome). Note that an expression pattern has been published for an *Acropora millepora* gene termed vnd*/nk2.1* (ref. 90 in Text S1), but *vnd* and the *Acropora* gene are orthologs of *nk2.2*. The maternally localized *frizzled* genes in *Clytia* are not orthologous to *frizzled5/8*.
(TIF)

Figure S2 *NvSix3/6* is expressed in some individual cells in the ecto- and endoderm. (A and B) In situ hybridizations with *NvSix3/6* probe at the planula stage, lateral views, aboral pole to the left in (A) and to the bottom in (B). Close-ups show expression in individual cells outside the main aboral expression domain (arrowheads). Scale bar, 100 µm.
(TIF)

Figure S3 Expression patterns of *NvFGFa2*, *NvSoxB(1)*, and *NvHoxF/Anthox1*. (A1–C4) In situ hybridizations with DIG-labelled probes; developmental stages are indicated on top, with probe on the left side. All three genes are expressed at the presumptive aboral side from the blastula stage on. Only the *NvSoxB(1)* signal is detectable already at the cleavage stage (A1–C1). *NvSoxB(1)* is also expressed in the pharynx (B3 and 4; [68]), and *NvHoxF/Anthox1* is expressed in scattered ectodermal cells in addition to the aboral pole (C4; [49]). Scale bar, 100 µm.
(TIF)

Figure S4 Morpholino control experiments. Overview images of gastrula embryos injected with the indicated morpholinos and mRNAs. mRNAs were synthesised from reporter constructs in which the morpholino target sites are cloned in front of the EGFP coding sequence. The gene-specific morpholinos block expression of their target (L–O) but not of control mRNAs (A–E). Control morpholino 2 does not affect expression of any mRNA (F–J). All images were acquired with identical settings, and the brightness of the whole figure was enhanced to make the gastrulae in (K–O) visible.
(TIF)

Figure S5 *NvFGFa1* represses *NvFoxD1* expression in the apical organ domain. In situ hybridizations with *NvFoxD1* probe at the planula stage, with lateral views with aboral pole to the left (A and B), aboral views in (A′ and B′); B′ is tilted sideways. *NvFoxD1* is a “ring gene,” since it is expressed aborally, with a gap in the apical organ region (A, A′; [63]). Injection of *NvFGFa1* MO suppresses the gap formation (B, B′). Scale bar, 100 µm.
(TIF)

Figure S6 Expression of *NvFoxD1* and *NvFGFRa* is regulated by *NvSix3/6*. In situ hybridizations at the planula stage, probes are indicated on the left side, with injected morpholinos at the top. (A, B, C, D) are lateral views, with aboral side to the left, and (A′, B′, C′, D′) are aboral views. (A–B′) The high-level expression of *NvFGFRa* at the aboral pole is absent in *NvSix3/6* MO-injected animals, but the low-level ectodermal expression persists. (C–D′) *NvFoxD1* expression is strongly reduced upon *NvSix3/6* MO injection. Scale bar, 100 µm. (C) and (C′) are the same images as Figure S5A and A′.
(TIF)

Table S1 Primer sequences for gene isolation.
(DOCX)

Table S2 Morpholino sequences.
(DOCX)

Table S3 Primer sequences for qPCR experiments.
(DOCX)

Text S1 References for Figure S1.
(DOCX)

## Abbreviations

A-P: anterior-posterior
DAPI: 4′,6-diamidino-2-phenylindole
DMSO: dimethyl sulfoxide
EGFP: enhanced green fluorescent protein
*FGF(R)*: *Fibroblast growth factor (receptor)*
FITC: fluorescein isothiocyanate
*fox*: *forkhead box*
GSK3: Glycogen Synthase Kinase 3
*hox*: *homeobox*
*irx*: *iroquois homeobox*
MO: morpholino
*Nv*: *Nematostella vectensis*
RACE: rapid amplification of cDNS ends
*rx*: *retinal homeobox*
*six*: *sine oculis homeobox homolog*
*sox*: *SRY (sex determing region Y) box*
UTP: uridine triphosphate
