# Supplementary figures and images for: The Bilaterian Head Patterning Gene six3/6 Controls Aboral Domain Development in a Cnidarian
Source: PLoS Biol. 2013 Feb 19;11(2):e1001488. doi: 10.1371/journal.pbio.1001488 (PMC3586664; doi:10.1371/journal.pbio.1001488)

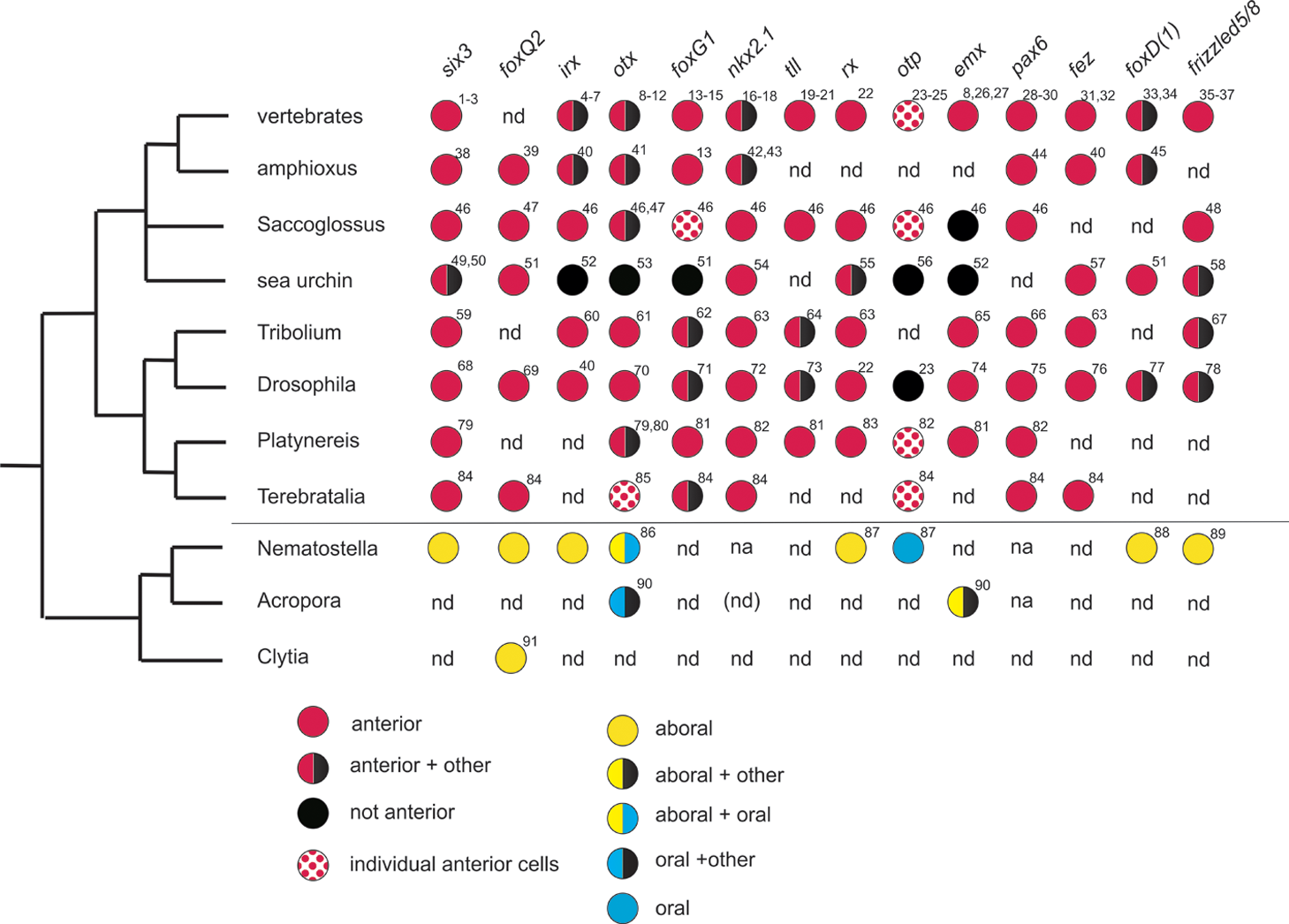

Supplement: Figure S1 — Summary of the expression of bilaterian anterior genes and their cnidarian orthologs. The color code for expression categories during embryonic and larval stages is shown at the bottom, with references at the top right of each circle. The list of references can be found in Text S1. Nd, not determined; na, not applicable (no clear ortholog present in genome). Note that an expression pattern has been published for an Acropora millepora gene termed vnd/nk2.1 (ref. 90 in Text S1), but vnd and the Acropora gene are orthologs of nk2.2. The maternally localized frizzled genes in Clytia are not orthologous to frizzled5/8. (TIF) [file pbio.1001488.s001.tif]

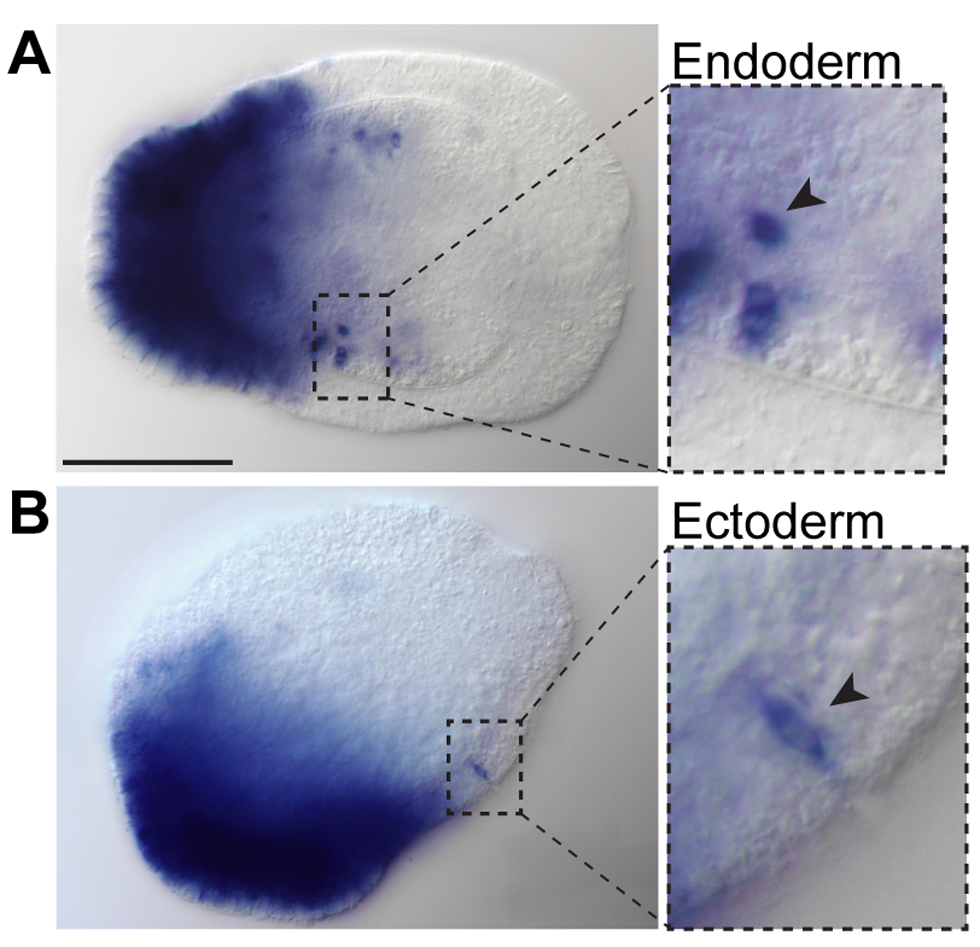

Supplement: Figure S2 — NvSix3/6 is expressed in some individual cells in the ecto- and endoderm. (A and B) In situ hybridizations with NvSix3/6 probe at the planula stage, lateral views, aboral pole to the left in (A) and to the bottom in (B). Close-ups show expression in individual cells outside the main aboral expression domain (arrowheads). Scale bar, 100 µm. (TIF) [file pbio.1001488.s002.tif]

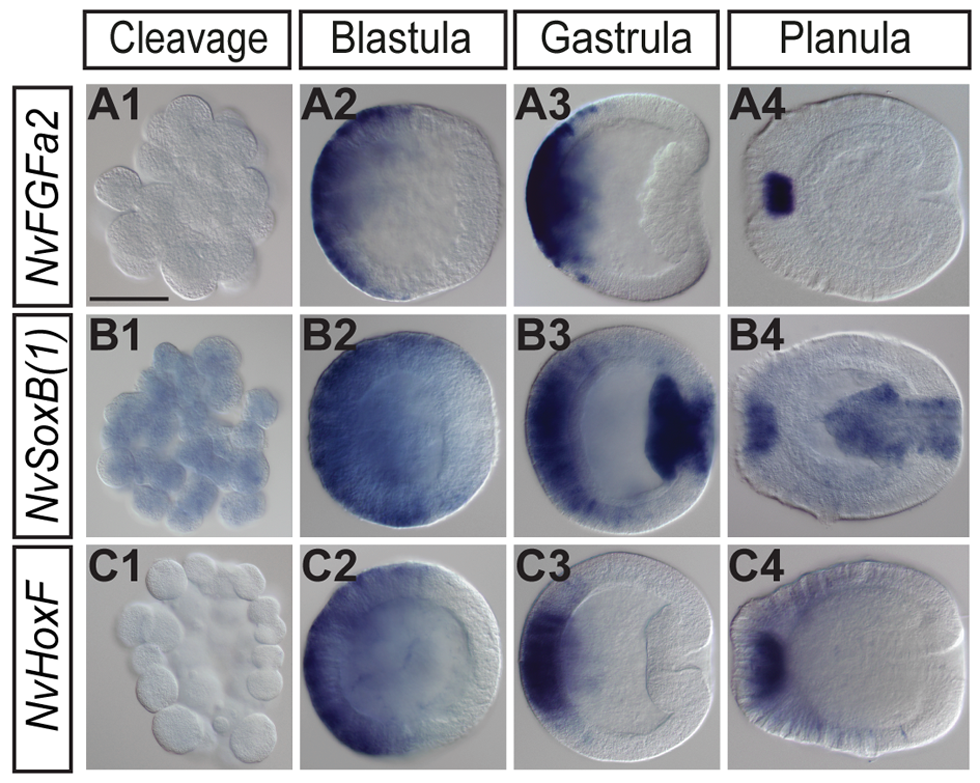

Supplement: Figure S3 — Expression patterns of NvFGFa2, NvSoxB(1), and NvHoxF/Anthox1. (A1–C4) In situ hybridizations with DIG-labelled probes; developmental stages are indicated on top, with probe on the left side. All three genes are expressed at the presumptive aboral side from the blastula stage on. Only the NvSoxB(1) signal is detectable already at the cleavage stage (A1–C1). NvSoxB(1) is also expressed in the pharynx (B3 and 4; [68]), and NvHoxF/Anthox1 is expressed in scattered ectodermal cells in addition to the aboral pole (C4; [49]). Scale bar, 100 µm. (TIF) [file pbio.1001488.s003.tif]

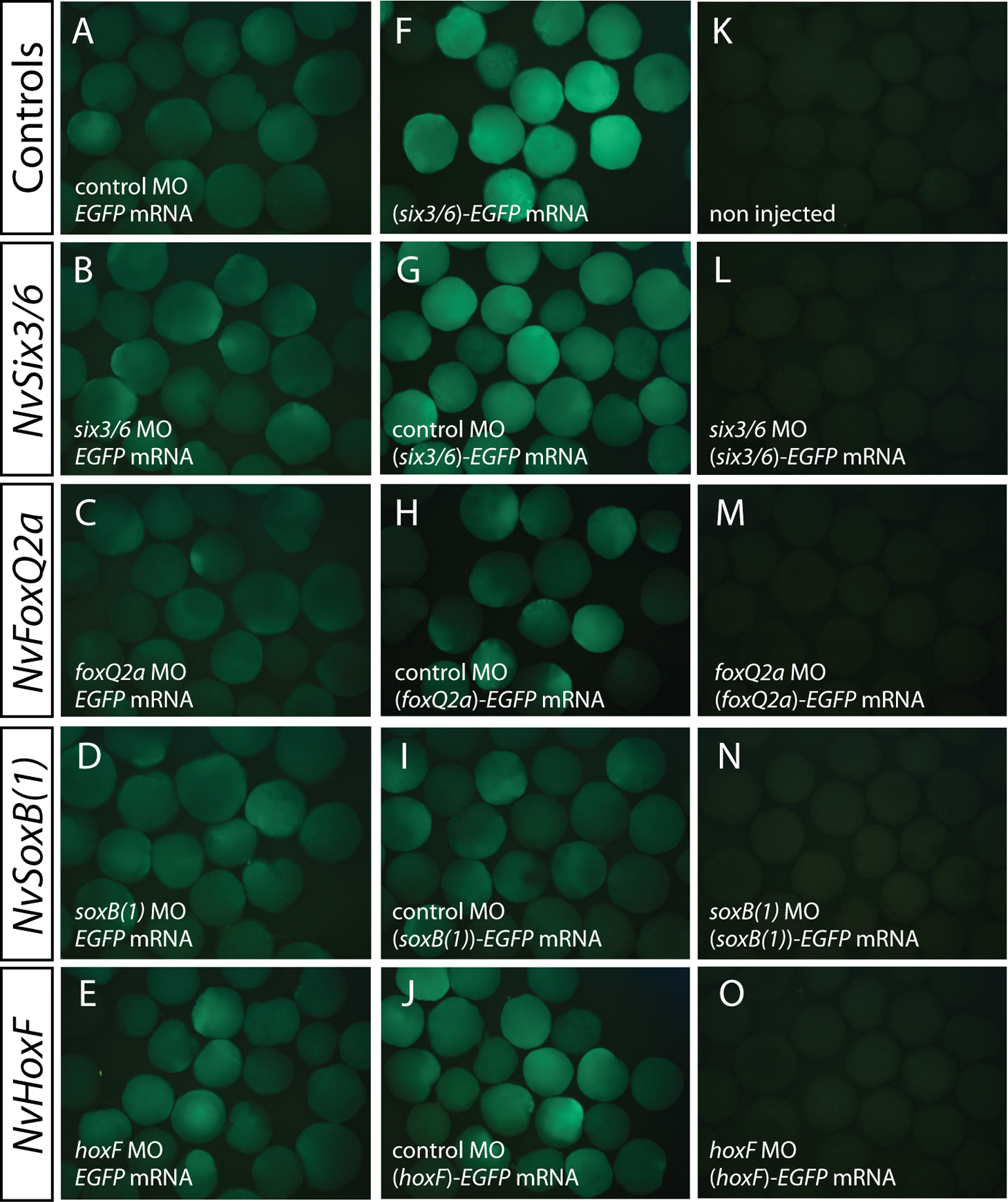

Supplement: Figure S4 — Morpholino control experiments. Overview images of gastrula embryos injected with the indicated morpholinos and mRNAs. mRNAs were synthesised from reporter constructs in which the morpholino target sites are cloned in front of the EGFP coding sequence. The gene-specific morpholinos block expression of their target (L–O) but not of control mRNAs (A–E). Control morpholino 2 does not affect expression of any mRNA (F–J). All images were acquired with identical settings, and the brightness of the whole figure was enhanced to make the gastrulae in (K–O) visible. (TIF) [file pbio.1001488.s004.tif]

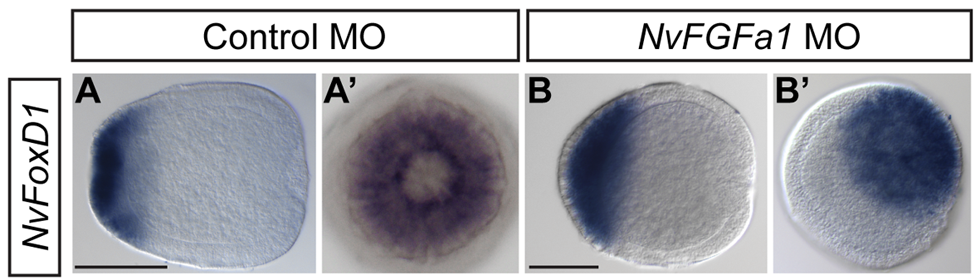

Supplement: Figure S5 — NvFGFa1 represses NvFoxD1 expression in the apical organ domain. In situ hybridizations with NvFoxD1 probe at the planula stage, with lateral views with aboral pole to the left (A and B), aboral views in (A′ and B′); B′ is tilted sideways. NvFoxD1 is a “ring gene,” since it is expressed aborally, with a gap in the apical organ region (A, A′; [63]). Injection of NvFGFa1 MO suppresses the gap formation (B, B′). Scale bar, 100 µm. (TIF) [file pbio.1001488.s005.tif]

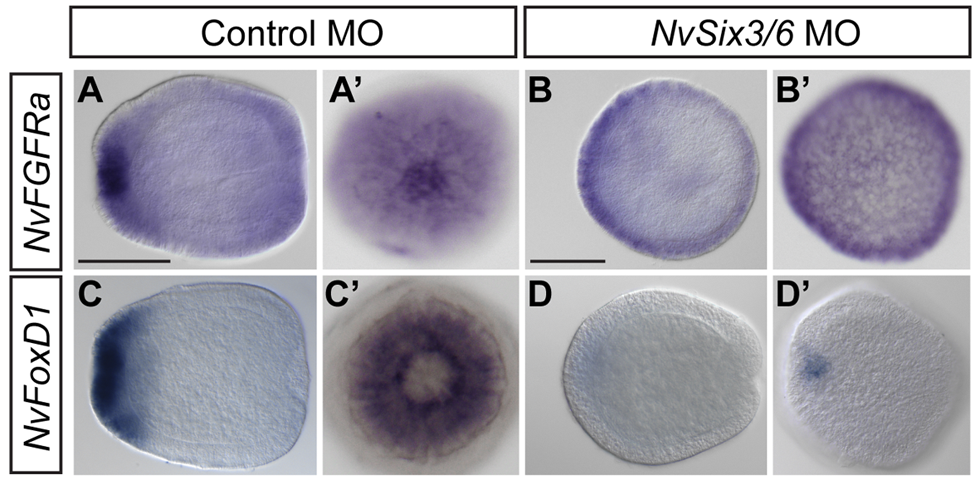

Supplement: Figure S6 — Expression of NvFoxD1 and NvFGFRa is regulated by NvSix3/6. In situ hybridizations at the planula stage, probes are indicated on the left side, with injected morpholinos at the top. (A, B, C, D) are lateral views, with aboral side to the left, and (A′, B′, C′, D′) are aboral views. (A–B′) The high-level expression of NvFGFRa at the aboral pole is absent in NvSix3/6 MO-injected animals, but the low-level ectodermal expression persists. (C–D′) NvFoxD1 expression is strongly reduced upon NvSix3/6 MO injection. Scale bar, 100 µm. (C) and (C′) are the same images as Figure S5A and A′. (TIF) [file pbio.1001488.s006.tif]
