# Supplementary material for: The Bilaterian Head Patterning Gene six3/6 Controls Aboral Domain Development in a Cnidarian
Source: PLoS Biol. 2013 Feb 19;11(2):e1001488. doi: 10.1371/journal.pbio.1001488 (PMC3586664; doi:10.1371/journal.pbio.1001488)
Supplement: Table S1 — Primer sequences for gene isolation. (DOCX) [file pbio.1001488.s007.docx]

**Table S1**

| **Primers for cloning of genes** (only unpublished genes) | |
| --- | --- |
| **six3/6-se** | CCATCCGCTTTAATAGAGTG |
| **six3/6-as** | CAGCCCAAAATACAAGCTACG |
| **six3/6_GSP1 (RACE)** | TCAAGCCACATCGCTTGCAGTTTAGCGTGC |
| **six3/6_nGSP1 (RACE)** | TGGGCGATTTGGTGTGCGCTAAAGCTG |
| **foxQ2a-se** | CAGAGCTAACCACCATGATGGC |
| **foxQ2a-as** | CAGTCGCTTTGTGTCTGGTG |
| **foxQ2a_GSP1 (RACE)** | AATGGCAGGAAATGCGGCGAACCAGGCGGG |
| **foxQ2a_nGSP1 (RACE)** | TGTTTCCATTCGAAATGGCGTGAAGGCGGA |
| **irx-se** | CTGAAGTGGTCCGAATTGAATC |
| **irx-as** | CTTCTGGTGCATTTTCCGTGGC |
| **foxJ1_GSP1 (RACE)** | CGCCAGAATCCGCCTTTTCCCGGTTCGTCC |
| **foxJ1_nGSP1 (RACE)** | GAGTTCTGCCAAGTCGGATCGGCAACTCTG |
| **foxJ1-se** | TTCAGAGAAAGAGGGAAGCCAGTC |
| **foxJ1-as** | ATTGAGTTCTGCCAAGTCGGATCG |
