# Supplementary material for: The Bilaterian Head Patterning Gene six3/6 Controls Aboral Domain Development in a Cnidarian
Source: PLoS Biol. 2013 Feb 19;11(2):e1001488. doi: 10.1371/journal.pbio.1001488 (PMC3586664; doi:10.1371/journal.pbio.1001488)
Supplement: Table S2 — Morpholino sequences. (DOCX) [file pbio.1001488.s008.docx]

**Table S2**

| **Morpholino** (sequences complementary to ATG are underlined) | |
| --- | --- |
| **Six3/6_UTR** | GTACTGCCGCACTGCAAGACTTGTC |
| **FoxQ2a_UTR** | GTTAGCTCTGGACTGTGCGACTACT |
| **FoxQ2a_ATG (2^nd^ Morpholino)** | TTGGTAAACAAGCAAATGCCATCAT |
| **HoxF_ATG** | TTGACTGCATGATGTGCGCTCTAGT |
| **SoxB1_ATG** | GCGTTAGAATAGTAGTAGCCGACAT |
| **Generic control MO** | CCATTTGAAGTTAAACgATAgATC |
| **Six3/6_mm** | GTAgTcCCGCAgTGCAAcACTTcTC |
