# Supplementary material for: The Bilaterian Head Patterning Gene six3/6 Controls Aboral Domain Development in a Cnidarian
Source: PLoS Biol. 2013 Feb 19;11(2):e1001488. doi: 10.1371/journal.pbio.1001488 (PMC3586664; doi:10.1371/journal.pbio.1001488)
Supplement: Table S3 — Primer sequences for qPCR experiments. (DOCX) [file pbio.1001488.s009.docx]

**Table S3**

| **Primers for qPCR** | |
| --- | --- |
| **ATPsynt_se_2** | TGCTGGGAAAGTTCTGGACCAATG |
| **ATPsynt_as_2** | ACACCCTCCTTGACGGTAACATTC |
| **EF1b_se_1** | TGCTGCATCAGAACAGAAACCTGC |
| **EF1b_as_1** | TAAGCCTTCAAGCGTTCTTGCCTG |
| **RibPrL23_se_1** | TTACGGAGCTCTGGCTTTCCTTTC |
| **RibPrL23_as_1** | TGCCGTTAAGGGTATCAAAGGACG |
| **six3/6_qPCR_se_1** | TTCTTTGGTCCTTGCCTGTGGC |
| **six3/6_qPCR_as_1** | TCGCTTGCAGTTTAGCGTGC |
| **foxQ2_qPCR_se_1** | CTGCCATTTCCACCATGTTACGCC |
| **foxQ2_qPCR_as_1** | GTTGGCCTGCATCTTGCTCTCTTC |
| **FGFa1_qPCR_se_1** | CTTCGAGATGCAATCATTCGGCCC |
| **FGFa1_qPCR_as_1** | TCGTCGTTCTAAGTGCACCGTCTC |
| **FGFa2_qPCR_se_1** | TCGAGGACGGAACCACTGATTGAG |
| **FGFa2_qPCR_as_1** | CACTGCGTAGAAAGATGTTGGCGG |
| **hoxF_qPCR_se_1** | GTTCGTTCCATCAGTACGCTTGGC |
| **hoxF_qPCR_as_1** | TCTCGTATACGCAGTCCTCTTGCG |
