# Supplementary material for: The Bilaterian Head Patterning Gene six3/6 Controls Aboral Domain Development in a Cnidarian
Source: PLoS Biol. 2013 Feb 19;11(2):e1001488. doi: 10.1371/journal.pbio.1001488 (PMC3586664; doi:10.1371/journal.pbio.1001488)
Supplement: Text S1 — References for Figure S1. (DOCX) [file pbio.1001488.s010.docx]

**TEXT S1**

1. Zhou X, Hollemann T, Pieler T, Gruss P (2000) Cloning and expression of xSix3, the Xenopus homologue of murine Six3. Mech Dev 91: 327-330.

2. Seo HC, Drivenes, Ellingsen S, Fjose A (1998) Expression of two zebrafish homologues of the murine Six3 gene demarcates the initial eye primordia. Mech Dev 73: 45-57.

3. Oliver G, Mailhos A, Wehr R, Copeland NG, Jenkins NA, et al. (1995) Six3, a murine homologue of the sine oculis gene, demarcates the most anterior border of the developing neural plate and is expressed during eye development. Development 121: 4045-4055.

4. Bosse A, Zulch A, Becker MB, Torres M, Gomez-Skarmeta JL, et al. (1997) Identification of the vertebrate Iroquois homeobox gene family with overlapping expression during early development of the nervous system. Mech Dev 69: 169-181.

5. Bellefroid EJ, Kobbe A, Gruss P, Pieler T, Gurdon JB, et al. (1998) Xiro3 encodes a Xenopus homolog of the Drosophila Iroquois genes and functions in neural specification. EMBO J 17: 191-203.

6. Gomez-Skarmeta JL, Glavic A, de la Calle-Mustienes E, Modolell J, Mayor R (1998) Xiro, a Xenopus homolog of the Drosophila Iroquois complex genes, controls development at the neural plate. EMBO J 17: 181-190.

7. Lecaudey V, Anselme I, Dildrop R, Ruther U, Schneider-Maunoury S (2005) Expression of the zebrafish Iroquois genes during early nervous system formation and patterning. J Comp Neurol 492: 289-302.

8. Simeone A, Acampora D, Gulisano M, Stornaiuolo A, Boncinelli E (1992) Nested expression domains of four homeobox genes in developing rostral brain. Nature 358: 687-690.

9. Tseng WF, Jang TH, Huang CB, Yuh CH (2011) An evolutionarily conserved kernel of gata5, gata6, otx2 and prdm1a operates in the formation of endoderm in zebrafish. Dev Biol 357: 541-557.

10. Chiao E, Leonard J, Dickinson K, Baker JC (2005) High-throughput functional screen of mouse gastrula cDNA libraries reveals new components of endoderm and mesoderm specification. Genome Res 15: 44-53.

11. Blitz IL, Cho KW (1995) Anterior neurectoderm is progressively induced during gastrulation: the role of the Xenopus homeobox gene orthodenticle. Development 121: 993-1004.

12. Pannese M, Polo C, Andreazzoli M, Vignali R, Kablar B, et al. (1995) The Xenopus homologue of Otx2 is a maternal homeobox gene that demarcates and specifies anterior body regions. Development 121: 707-720.

13. Toresson H, Martinez-Barbera JP, Bardsley A, Caubit X, Krauss S (1998) Conservation of BF-1 expression in amphioxus and zebrafish suggests evolutionary ancestry of anterior cell types that contribute to the vertebrate telencephalon. Dev Genes Evol 208: 431-439.

14. Tao W, Lai E (1992) Telencephalon-restricted expression of BF-1, a new member of the HNF-3/fork head gene family, in the developing rat brain. Neuron 8: 957-966.

15. Papalopulu N, Kintner C (1996) A posteriorising factor, retinoic acid, reveals that anteroposterior patterning controls the timing of neuronal differentiation in Xenopus neuroectoderm. Development 122: 3409-3418.

16. Rohr KB, Barth KA, Varga ZM, Wilson SW (2001) The nodal pathway acts upstream of hedgehog signaling to specify ventral telencephalic identity. Neuron 29: 341-351.

17. Sussel L, Marin O, Kimura S, Rubenstein JL (1999) Loss of Nkx2.1 homeobox gene function results in a ventral to dorsal molecular respecification within the basal telencephalon: evidence for a transformation of the pallidum into the striatum. Development 126: 3359-3370.

18. van den Akker WM, Brox A, Puelles L, Durston AJ, Medina L (2008) Comparative functional analysis provides evidence for a crucial role for the homeobox gene Nkx2.1/Titf-1 in forebrain evolution. J Comp Neurol 506: 211-223.

19. Hollemann T, Bellefroid E, Pieler T (1998) The Xenopus homologue of the Drosophila gene tailless has a function in early eye development. Development 125: 2425-2432.

20. Monaghan AP, Grau E, Bock D, Schutz G (1995) The mouse homolog of the orphan nuclear receptor tailless is expressed in the developing forebrain. Development 121: 839-853.

21. Kitambi SS, Hauptmann G (2007) The zebrafish orphan nuclear receptor genes nr2e1 and nr2e3 are expressed in developing eye and forebrain. Gene Expr Patterns 7: 521-528.

22. Mathers PH, Grinberg A, Mahon KA, Jamrich M (1997) The Rx homeobox gene is essential for vertebrate eye development. Nature 387: 603-607.

23. Simeone A, D'Apice MR, Nigro V, Casanova J, Graziani F, et al. (1994) Orthopedia, a novel homeobox-containing gene expressed in the developing CNS of both mouse and Drosophila. Neuron 13: 83-101.

24. Ryu S, Mahler J, Acampora D, Holzschuh J, Erhardt S, et al. (2007) Orthopedia homeodomain protein is essential for diencephalic dopaminergic neuron development. Curr Biol 17: 873-880.

25. Dominguez L, Gonzalez A, Moreno N (2010) Sonic hedgehog expression during Xenopus laevis forebrain development. Brain Res 1347: 19-32.

26. Morita T, Nitta H, Kiyama Y, Mori H, Mishina M (1995) Differential expression of two zebrafish emx homeoprotein mRNAs in the developing brain. Neurosci Lett 198: 131-134.

27. Pannese M, Lupo G, Kablar B, Boncinelli E, Barsacchi G, et al. (1998) The Xenopus Emx genes identify presumptive dorsal telencephalon and are induced by head organizer signals. Mech Dev 73: 73-83.

28. Hirsch N, Harris WA (1997) Xenopus Pax-6 and retinal development. J Neurobiol 32: 45-61.

29. Stoykova A, Gruss P (1994) Roles of Pax-genes in developing and adult brain as suggested by expression patterns. J Neurosci 14: 1395-1412.

30. Amirthalingam K, Lorens JB, Saetre BO, Salaneck E, Fjose A (1995) Embryonic expression and DNA-binding properties of zebrafish pax-6. Biochem Biophys Res Commun 215: 122-128.

31. Matsuo-Takasaki M, Lim JH, Beanan MJ, Sato SM, Sargent TD (2000) Cloning and expression of a novel zinc finger gene, Fez, transcribed in the forebrain of Xenopus and mouse embryos. Mech Dev 93: 201-204.

32. Hashimoto H, Yabe T, Hirata T, Shimizu T, Bae Y, et al. (2000) Expression of the zinc finger gene fez-like in zebrafish forebrain. Mech Dev 97: 191-195.

33. Hatini V, Tao W, Lai E (1994) Expression of winged helix genes, BF-1 and BF-2, define adjacent domains within the developing forebrain and retina. J Neurobiol 25: 1293-1309.

34. Gomez-Skarmeta JL, de la Calle-Mustienes E, Modolell J, Mayor R (1999) Xenopus brain factor-2 controls mesoderm, forebrain and neural crest development. Mech Dev 80: 15-27.

35. Burns CJ, Zhang J, Brown EC, Van Bibber AM, Van Es J, et al. (2008) Investigation of Frizzled-5 during embryonic neural development in mouse. Dev Dyn 237: 1614-1626.

36. Deardorff MA, Tan C, Conrad LJ, Klein PS (1998) Frizzled-8 is expressed in the Spemann organizer and plays a role in early morphogenesis. Development 125: 2687-2700.

37. Kim SH, Park HC, Yeo SY, Hong SK, Choi JW, et al. (1998) Characterization of two frizzled8 homologues expressed in the embryonic shield and prechordal plate of zebrafish embryos. Mech Dev 78: 193-201.

38. Kozmik Z, Holland ND, Kreslova J, Oliveri D, Schubert M, et al. (2007) Pax-Six-Eya-Dach network during amphioxus development: conservation in vitro but context specificity in vivo. Dev Biol 306: 143-159.

39. Yu JK, Holland ND, Holland LZ (2003) AmphiFoxQ2, a novel winged helix/forkhead gene, exclusively marks the anterior end of the amphioxus embryo. Dev Genes Evol 213: 102-105.

40. Irimia M, Pineiro C, Maeso I, Gomez-Skarmeta JL, Casares F, et al. (2010) Conserved developmental expression of Fezf in chordates and Drosophila and the origin of the Zona Limitans Intrathalamica (ZLI) brain organizer. Evodevo 1: 7.

41. Schubert M, Yu JK, Holland ND, Escriva H, Laudet V, et al. (2005) Retinoic acid signaling acts via Hox1 to establish the posterior limit of the pharynx in the chordate amphioxus. Development 132: 61-73.

42. Venkatesh TV, Holland ND, Holland LZ, Su MT, Bodmer R (1999) Sequence and developmental expression of amphioxus AmphiNk2-1: insights into the evolutionary origin of the vertebrate thyroid gland and forebrain. Dev Genes Evol 209: 254-259.

43. Ogasawara M (2000) Overlapping expression of amphioxus homologs of the thyroid transcription factor-1 gene and thyroid peroxidase gene in the endostyle: insight into evolution of the thyroid gland. Dev Genes Evol 210: 231-242.

44. Glardon S, Holland LZ, Gehring WJ, Holland ND (1998) Isolation and developmental expression of the amphioxus Pax-6 gene (AmphiPax-6): insights into eye and photoreceptor evolution. Development 125: 2701-2710.

45. Yu JK, Holland ND, Holland LZ (2002) An amphioxus winged helix/forkhead gene, AmphiFoxD: insights into vertebrate neural crest evolution. Dev Dyn 225: 289-297.

46. Lowe CJ, Wu M, Salic A, Evans L, Lander E, et al. (2003) Anteroposterior patterning in hemichordates and the origins of the chordate nervous system. Cell 113: 853-865.

47. Darras S, Gerhart J, Terasaki M, Kirschner M, Lowe CJ (2011) beta-catenin specifies the endomesoderm and defines the posterior organizer of the hemichordate Saccoglossus kowalevskii. Development 138: 959-970.

48. Pani AM, Mullarkey EE, Aronowicz J, Assimacopoulos S, Grove EA, et al. (2012) Ancient deuterostome origins of vertebrate brain signalling centres. Nature 483: 289-294.

49. Poustka AJ, Kuhn A, Groth D, Weise V, Yaguchi S, et al. (2007) A global view of gene expression in lithium and zinc treated sea urchin embryos: new components of gene regulatory networks. Genome Biol 8: R85.

50. Wei Z, Yaguchi J, Yaguchi S, Angerer RC, Angerer LM (2009) The sea urchin animal pole domain is a Six3-dependent neurogenic patterning center. Development 136: 1179-1189.

51. Tu Q, Brown CT, Davidson EH, Oliveri P (2006) Sea urchin Forkhead gene family: phylogeny and embryonic expression. Dev Biol 300: 49-62.

52. Howard-Ashby M, Materna SC, Brown CT, Chen L, Cameron RA, et al. (2006) Identification and characterization of homeobox transcription factor genes in Strongylocentrotus purpuratus, and their expression in embryonic development. Dev Biol 300: 74-89.

53. Li X, Chuang CK, Mao CA, Angerer LM, Klein WH (1997) Two Otx proteins generated from multiple transcripts of a single gene in Strongylocentrotus purpuratus. Dev Biol 187: 253-266.

54. Takacs CM, Amore G, Oliveri P, Poustka AJ, Wang D, et al. (2004) Expression of an NK2 homeodomain gene in the apical ectoderm defines a new territory in the early sea urchin embryo. Dev Biol 269: 152-164.

55. Burke RD, Angerer LM, Elphick MR, Humphrey GW, Yaguchi S, et al. (2006) A genomic view of the sea urchin nervous system. Dev Biol 300: 434-460.

56. Di Bernardo M, Castagnetti S, Bellomonte D, Oliveri P, Melfi R, et al. (1999) Spatially restricted expression of PlOtp, a Paracentrotus lividus orthopedia-related homeobox gene, is correlated with oral ectodermal patterning and skeletal morphogenesis in late-cleavage sea urchin embryos. Development 126: 2171-2179.

57. Yaguchi S, Yaguchi J, Wei Z, Jin Y, Angerer LM, et al. (2011) Fez function is required to maintain the size of the animal plate in the sea urchin embryo. Development 138: 4233-4243.

58. Croce J, Duloquin L, Lhomond G, McClay DR, Gache C (2006) Frizzled5/8 is required in secondary mesenchyme cells to initiate archenteron invagination during sea urchin development. Development 133: 547-557.

59. Posnien N, Bashasab F, Bucher G (2009) The insect upper lip (labrum) is a nonsegmental appendage-like structure. Evol Dev 11: 480-488.

60. Posnien N, Bucher G (2010) Formation of the insect head involves lateral contribution of the intercalary segment, which depends on Tc-labial function. Dev Biol 338: 107-116.

61. Li Y, Brown S, Hausdorf B, Tautz D, Denell R, et al. (1996) Two orthodenticle-related genes in the short-germ beetle Tribolium castaneum. Development, Genes and Evolution 206: 35-45.

62. Choe CP, Miller SC, Brown SJ (2006) A pair-rule gene circuit defines segments sequentially in the short-germ insect Tribolium castaneum. Proc Natl Acad Sci U S A 103: 6560-6564.

63. Posnien N, Koniszewski ND, Hein HJ, Bucher G (2011) Candidate gene screen in the red flour beetle Tribolium reveals six3 as ancient regulator of anterior median head and central complex development. PLoS Genet 7: e1002416.

64. Schroder R, Eckert C, Wolff C, Tautz D (2000) Conserved and divergent aspects of terminal patterning in the beetle Tribolium castaneum. Proc Natl Acad Sci U S A 97: 6591-6596.

65. Schinko JB, Kreuzer N, Offen N, Posnien N, Wimmer EA, et al. (2008) Divergent functions of orthodenticle, empty spiracles and buttonhead in early head patterning of the beetle Tribolium castaneum (Coleoptera). Dev Biol 317: 600-613.

66. Yang X, Weber M, Zarinkamar N, Posnien N, Friedrich F, et al. (2009) Probing the Drosophila retinal determination gene network in Tribolium (II): The Pax6 genes eyeless and twin of eyeless. Dev Biol 333: 215-227.

67. Beermann A, Pruhs R, Lutz R, Schroder R (2011) A context-dependent combination of Wnt receptors controls axis elongation and leg development in a short germ insect. Development 138: 2793-2805.

68. Seimiya M, Gehring WJ (2000) The Drosophila homeobox gene optix is capable of inducing ectopic eyes by an eyeless-independent mechanism. Development 127: 1879-1886.

69. Lee HH, Frasch M (2004) Survey of forkhead domain encoding genes in the Drosophila genome: Classification and embryonic expression patterns. Dev Dyn 229: 357-366.

70. Finkelstein R, Perrimon N (1990) The orthodenticle gene is regulated by bicoid and torso and specifies Drosophila head development. Nature 346: 485-488.

71. Grossniklaus U, Pearson RK, Gehring WJ (1992) The Drosophila sloppy paired locus encodes two proteins involved in segmentation that show homology to mammalian transcription factors. Genes Dev 6: 1030-1051.

72. Zaffran S, Das G, Frasch M (2000) The NK-2 homeobox gene scarecrow (scro) is expressed in pharynx, ventral nerve cord and brain of Drosophila embryos. Mech Dev 94: 237-241.

73. Pignoni F, Baldarelli RM, Steingrimsson E, Diaz RJ, Patapoutian A, et al. (1990) The Drosophila gene tailless is expressed at the embryonic termini and is a member of the steroid receptor superfamily. Cell 62: 151-163.

74. Dalton D, Chadwick R, McGinnis W (1989) Expression and embryonic function of empty spiracles: a Drosophila homeo box gene with two patterning functions on the anterior-posterior axis of the embryo. Genes Dev 3: 1940-1956.

75. Quiring R, Walldorf U, Kloter U, Gehring WJ (1994) Homology of the eyeless gene of Drosophila to the Small eye gene in mice and Aniridia in humans. Science 265: 785-789.

76. Pfeiffer BD, Jenett A, Hammonds AS, Ngo TT, Misra S, et al. (2008) Tools for neuroanatomy and neurogenetics in Drosophila. Proc Natl Acad Sci U S A 105: 9715-9720.

77. Hacker U, Grossniklaus U, Gehring WJ, Jackle H (1992) Developmentally regulated Drosophila gene family encoding the fork head domain. Proc Natl Acad Sci U S A 89: 8754-8758.

78. Bhanot P, Brink M, Samos CH, Hsieh JC, Wang Y, et al. (1996) A new member of the frizzled family from Drosophila functions as a Wingless receptor. Nature 382: 225-230.

79. Steinmetz PR, Urbach R, Posnien N, Eriksson J, Kostyuchenko RP, et al. (2010) Six3 demarcates the anterior-most developing brain region in bilaterian animals. Evodevo 1: 14.

80. Arendt D, Technau U, Wittbrodt J (2001) Evolution of the bilaterian larval foregut. Nature 409: 81-85.

81. Tomer R, Denes AS, Tessmar-Raible K, Arendt D (2010) Profiling by image registration reveals common origin of annelid mushroom bodies and vertebrate pallium. Cell 142: 800-809.

82. Tessmar-Raible K, Raible F, Christodoulou F, Guy K, Rembold M, et al. (2007) Conserved sensory-neurosecretory cell types in annelid and fish forebrain: insights into hypothalamus evolution. Cell 129: 1389-1400.

83. Arendt D, Tessmar-Raible K, Snyman H, Dorresteijn AW, Wittbrodt J (2004) Ciliary photoreceptors with a vertebrate-type opsin in an invertebrate brain. Science 306: 869-871.

84. Santagata S, Resh C, Hejnol A, Martindale MQ, Passamaneck YJ (2012) Development of the larval anterior neurogenic domains of Terebratalia transversa (Brachiopoda) provides insights into the diversification of larval apical organs and the spiralian nervous system. Evodevo 3: 3.

85. Passamaneck YJ, Furchheim N, Hejnol A, Martindale MQ, Luter C (2011) Ciliary photoreceptors in the cerebral eyes of a protostome larva. Evodevo 2: 6.

86. Mazza ME, Pang K, Martindale MQ, Finnerty JR (2007) Genomic organization, gene structure, and developmental expression of three clustered otx genes in the sea anemone Nematostella vectensis. J Exp Zool B Mol Dev Evol 308: 494-506.

87. Mazza ME, Pang K, Reitzel AM, Martindale MQ, Finnerty JR (2010) A conserved cluster of three PRD-class homeobox genes (homeobrain, rx and orthopedia) in the Cnidaria and Protostomia. Evodevo 1: 3.

88. Magie CR, Pang K, Martindale MQ (2005) Genomic inventory and expression of Sox and Fox genes in the cnidarian Nematostella vectensis. Dev Genes Evol 215: 618-630.

89. Kumburegama S, Wijesena N, Xu R, Wikramanayake AH (2011) Strabismus-mediated primary archenteron invagination is uncoupled from Wnt/beta-catenin-dependent endoderm cell fate specification in Nematostella vectensis (Anthozoa, Cnidaria): Implications for the evolution of gastrulation. Evodevo 2: 2.

90. de Jong DM, Hislop NR, Hayward DC, Reece-Hoyes JS, Pontynen PC, et al. (2006) Components of both major axial patterning systems of the Bilateria are differentially expressed along the primary axis of a 'radiate' animal, the anthozoan cnidarian Acropora millepora. Dev Biol 298: 632-643.

91. Chevalier S, Martin A, Leclere L, Amiel A, Houliston E (2006) Polarised expression of FoxB and FoxQ2 genes during development of the hydrozoan Clytia hemisphaerica. Dev Genes Evol 216: 709-720.
